# ANARCII enables alignment-free antigen receptor numbering using a generalised language model

**DOI:** 10.1038/s42003-026-10186-z

**Published:** 2026-05-21

**Authors:** Alexander Greenshields-Watson, Parth Agarwal, Sarah A. Robinson, Benjamin Heathcote Williams, Gemma L. Gordon, Henriette L. Capel, Yushi Li, Fabian C. Spoendlin, Broncio Aguilar-Sanjuan, Fergus Boyles, Charlotte M. Deane

**Affiliations:** Department of Statistics, Oxford Protein Informatics Group, University of Oxford, Oxford, UK

**Keywords:** Data processing, Sequence annotation, VDJ recombination, Somatic hypermutation

## Abstract

Antigen receptor numbering allows delineation of antigen-binding regions of antibodies and T cell receptors, from sequence alone. Numbering is currently achieved by aligning to a reference set. This approach may result in different numbering depending on reference set used or fail on sequences from rare species or formats. We present a method (ANARCII) which requires no alignment step and is based on a Seq2Seq language model. ANARCII improves upon existing methods through more consistent numbering of key regions, robustness to truncations, generalisation to unseen species, and easier user installation. The lightweight architecture allows numbering of 90,000 sequences per minute on a high-end GPU. The software is available via web app (https://opig.stats.ox.ac.uk/webapps/sabdab-sabpred/sabpred/anarcii/), and package (https://github.com/oxpig/ANARCII). Ultimately ANARCII allows numbering of more antibody-like sequences, with better recovery of full-length regions from existing databases, and enables comparative analysis of new receptors not numbered by existing tools.

## Abstract (web summary)

ANARCII utilises a language model approach to enable alignment free numbering of antigen receptors with improved consistency over existing methods as well as generalisation to novel species and sequence formats.

## Introduction

Standard antibodies are Y-shaped proteins made up of two pairs of identical heavy and light chains. Their ability to bind to target epitopes with high affinity and specificity makes them highly attractive therapeutics^1^. Antibody structure can be divided into the fragment antigen-binding (Fab) region, comprising the entire light chain together with the variable and first constant (CH1) domains of the heavy chain, and the Fc region, formed by the remaining constant domains of the heavy chain. The number of constant domains varies between antibody classes. The distal domains of the Fab (VH and VL) form the variable region (Fv) on which are found the complementarity-determining region loops (CDR1, CDR2 and CDR3), the main segments of an antibody involved in antigen binding.

In antibody heavy chains, sequence diversity arises from the assembly of variable (V), diversity (D) and joining (J) gene segments to form a complete chain^2^, in a process termed VDJ recombination. The genome of an individual contains multiple distinct V, D and J genes; the steps and enzymes involved in joining these genes introduce random mutations giving rise to junctional diversity^3^. The junctions are situated in the CDR3 region of the heavy chain (CDRH3), leading to this loop being the focus of maximal sequence diversity and a primary driver of antigen recognition^4^. Additionally, when an antibody undergoes affinity maturation to a target antigen, the process of somatic hypermutation (SHM) introduces further random mutations along the sequence^5^. Mutations which result in higher affinity for the target are positively selected and may dominate the immune response. In light chains, the process is identical, except for the absence of a D gene in the generation process.

Applying numbering schemes to the antibody Fv region provides a way to navigate the vast diversity that these mechanisms generate, and to compare many sequences using a consistent frame of reference. The substitutions, insertions and deletions that arise in SHM can be identified and numbered with respect to the original parent germline gene sequence. The primary purpose of numbering an Fv region is to delineate the start and end of the CDR loops from the beta sheet framework regions (FR) from which they protrude^6^. There are several different schemes which are used to number antibodies. The earliest suggested scheme is the Kabat scheme^7^. This was created without any structural information and using only a small number of sequences. More structurally aware derivates of the Kabat scheme, Chothia^8^ and Martin^9^ followed. Further developments included the Aho scheme^10^ which sought to be highly representative of structure, and the Wolfguy system^11^ which allowed compliance with docking tools that do not accept repeated use of the same number (a problem when docking heavy and light chains). The most widely used numbering scheme is IMGT^12^. First designed in 1997^13^, the scheme has consistent placement of conserved residues and regions across antibody heavy and light chains, as well as TCR alpha, beta, gamma and delta chains. As it utilises the germline reference sequences as the basis for numbering, insertions and deletions can be identified with respect to the parent allele. It is conceptually simple to understand, and the structural equivalence of numbering positions makes it easy to compare across sequence types, chains, genes and alleles.

Accurate antibody numbering relies on the correct identification of highly conserved residues that are crucial to the structure of the immunoglobulin fold and help define framework and CDR boundaries. In particular, conserved cysteine residues in the variable domain (e.g. positions 23 and 104 in IMGT numbering) form the canonical intradomain disulfide bonds essential for structural stability, while conserved residues such as tryptophan at position 41 contribute to the hydrophobic core of the immunoglobulin fold^8^. Misidentification of these residues leads to incorrect framework–CDR segmentation and downstream structural and functional misinterpretation. For this reason, accurate detection of conserved positions provides a sensitive and biologically meaningful benchmark for evaluating antibody numbering methods.

Currently, antibodies are numbered by aligning the sequence of an antibody to either a consensus sequence or the germline reference sequences. Tools including AbRSA^14^ and AntPack^15^ align to a consensus sequence using BLOSUM62^16^, custom gap penalties and the Needleman–Wunsch algorithm^17^. IMGT V-quest^18^ and IgBLAST^19^ use Smith-Waterman^20^ or BLAST^21^, respectively to align against IMGT reference sequences and return the highest scoring alignment. An alternative approach is used by ANARCI (pronounced ‘anarchy’)^22^, which scores a query sequence against multiple Hidden Markov Models (HMMs) built on sets of germline sequences for a given species and chain.

All these methods rely on either a representative consensus sequence or an IMGT germline reference set compiled using data available at a specific point in time. Germline references are continually being updated and modified as new species, genes, alleles and sequence types are discovered^23,24^. This means the ground truth needed to number an antibody is not static, and the numbering returned may differ depending on the timestamp of the reference set.

For example, each time a user installs ANARCI, they are required to compile the HMM models for all reference germlines in IMGT. A single master HMM cannot be created, so multiple models are built and each is used to score an input query sequence with the alignment to the highest scoring reference returned. Small changes in the reference sets, e.g. the addition of new alleles to IMGT data, can lead to alternate predictions which can lead to large differences in numbering, including changes to CDR delineation. Furthermore, the addition of new species to ANARCI reference sets, for example, to enable numbering of Rhesus Macaques, can in some cases corrupt the numbering of human sequences due to alignment to a highly similar but erroneous reference.

Users of ANARCI have repeatedly identified such differences in numbering depending on when the HMM models were compiled (see GitHub issues #40 and #64 at https://github.com/oxpig/ANARCI). These differences are frequently observed in sequences with long CDRH3s, or those with truncations, where several residues are missing from the start (before the CDR1), or the end (close to the CDR3). The HMM models are particularly sensitive to these scenarios, and small differences in reference sets often result in alternate numberings. Truncations are a particularly common feature of NGS data and have been found to occur in up to 40% of sequences taken from Observed Antibody Space (OAS)^25–27^.

An additional problem is that HMM compilation requires an organisation such as IMGT to host and continually update these germline reference sets and maintain a format workable with ANARCI’s dependencies. When the IMGT website is no longer available (see GitHub issue #97), users are unable to install ANARCI from source and must rely on historic builds, which often diverge from recent builds.

Furthermore, query sequences which originate from rare species or uncommon sequence formats (e.g. novel single-chain constructs) are more challenging to number with current approaches, which rely on static sequence representations. This is because the sequences can be very different from the data used to construct the consensus sequences or HMMs, and the existing methods cannot generalise to novel sequence types. This problem is becoming increasingly relevant as more antibody sequence data from new species, alleles and sequence types is deposited in the public domain^24^. One example of this is shark variable domain of new antigen receptor (VNAR) antibodies^28^, where the large numbers of proximal cysteines, as well as non-standard CDRH2 loop placement, mean that existing tools cannot provide numbering appropriate for their unique structural morphology^29^.

Large language models have been successful in many protein sequence/structure tasks^30,31^, suggesting that they may capture the sequence patterns which relate to structure. In this paper, we explore whether a language model^32^ can accurately number antigen receptors and effectively generalise to new sequence types and patterns.

By training a model on a highly diverse set of numbered sequences, we hoped to capture the key unifying patterns evident and thus move away from the need to revert to manually curated reference sets when faced with new alleles, species or novel formats.

Models were trained on over 160M antibody heavy and light chain sequences. Each sequence was augmented with random, non-antibody sequence content. During training models were tasked with distinguishing antibody sequence content from non-antibody content, predicting the correct chain identity (heavy, kappa or lambda) and applying IMGT numbering to the sequence. Multiple model architectures were investigated to find the optimum balance of inference speed and numbering accuracy. The final model suite, ANARCII (pronounced ‘anarchy two’), showed highly consistent numbering, whilst generalising to sequence types which could not previously be numbered in any form, including VNARs and highly unconventional antibody-like sequences. When challenged with ambiguous sequences containing truncations and long CDR3s, we found ANARCII outperformed existing tools in the identification of conserved residues and complete CDR regions.

Furthermore, the unique flexibility in label prediction by a language model allows for ANARCII outputs to be conditioned to adapt predicted numbering to highly novel sequence types. We exploited this to number 456 shark VNAR sequences with unconventional sequence features so that they fully matched the IMGT definition around the CDR2. A version of the ANARCII model fine-tuned on this renumbered VNAR dataset could accurately predict the correct numbering on held-out VNAR test sequences without conditioning and enabled the first comparative analysis of VNAR and VHH sequences^33^. We also demonstrate that the ANARCII antibody models with only minimal fine-tuning can accurately number T cell receptor (TCR) sequences. Multiple model types are made accessible to users in the ANARCII software suite, as well as instructions on how to condition auto-regressive outputs to create custom schemes and workflows. ANARCII will be increasingly useful to antibody researchers and engineers as they navigate the vast landscape of antibody sequence space. It also serves as a proof of concept that small language models can be effective in sequence alignment tasks, offering a flexible alternative to current methods.

## Results

### Training a Seq2Seq Language model to number antigen receptor sequences

**Fig. 1:**
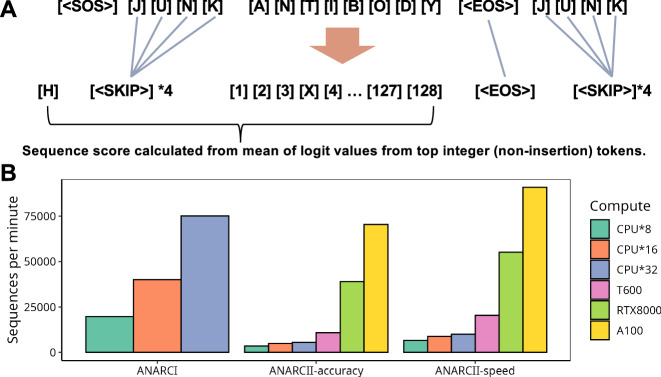
ANARCII is a Seq2Seq language model trained to identify chain types and number sequences. Schematic of sequence to number training task (**A**), where input sequences are bookended by random amino acids, which during training must be assigned the corresponding [<SKIP>] token. In addition, each target sequence has a chain token which the model is trained to predict before assigning numbering. **B** Comparison of inference speeds on 100,000 sequences across different CPU and GPU architectures for ANARCI, and the ANARCII models.

In order to develop a model that could ‘translate’ the amino acid sequences of antigen receptors into their corresponding IMGT number labels, we utilised the Seq2Seq transformer architecture and the neural machine translation task. Our model, ANARCII, is designed to both number and call the chain (heavy, kappa or lambda) of a given antibody Fv sequence. Figure 1A shows a schematic of the training task, in which the model learns to predict [<CHAIN>], numbering (IMGT numbers 1–128) and insertion [<X>] tokens from an input amino acid sequence (Fig. 1A, and Supplementary Fig. 1). Sequences found in the PDB^34^ or in experimental data^25^ often contain extra content such as leader sequences. Early experiments involved testing the ability of a language model to predict the numbering of isolated VH domains without any extra content. These models were highly successful in numbering of clean VH sequences, but failed to accurately number when any non-antibody sequence content such as linkers or leader sequences were present. Therefore, we chose to adopt a data augmentation approach using random non-antibody sequence content termed ‘junk’ to our train, validation and test sequences. To ensure that ANARCII can correctly number experimental data of this type, we randomly added between 0 and 40 amino acids to the start and end of every sequence during training (termed ‘junk’ in Fig. 1A) and trained the model to output a [<SKIP>] token as the prediction, with the [<EOS>] token directly after the antibody sequence content.

As described in the methods, the training data were ~95.7 M heavy, ~41.6 M kappa and ~33.4 M lambda non-redundant sequences sampled from the OAS. Heavy, kappa and lambda sequences were each clustered based on sequence identity, then the resulting clusters split into train, test and validation sets (90:5:5, see ‘Methods’, Supplementary Fig. 2, Supplementary Tables 1 and 2, and Supplementary Note 1). Hyperparameters for ANARCII were chosen based on the validation set accuracy and the corresponding inference speed (Supplementary Table 3). While large parameter models show the best validation loss, often the difference in numbering accuracy was very small (differing between 0.1 and 0.2%) and did not justify the increases in inference time associated with having more parameters. We created two versions of ANARCII: ANARCII-accuracy and ANARCII-speed. The original ANARCI (without calling V/J gene usage) can number ~75,000 sequences per minute (SPM) using 32 CPUs. ANARCII-accuracy on an A100 GPU can number ~70,000 SPM, while ANARCII-speed can number ~90,000 SPM on the same architecture (Fig. 1B).

### ANARCII models can consistently and accurately IMGT number antigen receptor sequences

**Fig. 2:**
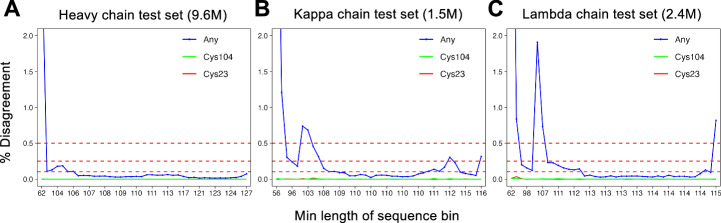
Sequence with truncations are the main source of disagreement between HMM (ANARCI) and LM (ANARCII-accuracy) numbering. Disagreement in numbering of ANARCI and ANARCII-accuracy versus sequence length on held out test sets of heavy (**A**), kappa (**B**) and lambda (**C**) chains. Test sequences are divided into 40 bins based on length, indicated on the *x* axes, from short to longer sequences.

To investigate the accuracy of our ANARCII models, we used them to number the held-out test sets of ~9.6M heavy, ~1.5M kappa and ~2.4M lambda sequences and compared the results against ANARCI. These test sets included sequences of varying length with many start or end truncations, typically observed in NGS datasets. ANARCII-speed exactly replicated the ANARCI numbering on 99.82% (heavy), 99.59% (kappa) and 99.54% (lambda) of the respective test sets (Table 1), while ANARCII-accuracy agreed on 99.87%, 99.67% and 99.62%. We next explored where the numbering predictions diverged between ANARCI and ANARCII and found that most disagreements were at the ends of truncated test sequences where identification of the first or last residues was not clear, and the remainder of the sequence numbering was identical (far left region of plots in Fig. 2A–C). We also found that most divergence, especially in light chains, occurred in sequences which did not contain conserved residues such as Cys104 or Trp41.

**Table 1:** ANARCII models performance on held out test sequences (including truncated sequences). % Agreement with ANARCI

Test set	Model	Overall	CYS-23	CYS-104	TRP-41	Ar-118	CDR1	CDR2	CDR3	DE-Loop
Heavy	Speed	99.8233	99.9999	99.9992	99.9999	99.9969	99.9898	99.9578	99.9747	99.9826
Heavy comp		99.9545	99.9999	99.9994	99.9999	99.9971	99.9947	99.9619	99.9895	99.9839
Heavy junk		96.095	99.9986	99.9964	99.9908	99.9943	99.8105	99.6688	99.7777	99.9126
Kappa		99.5937	99.9982	99.9936	99.9974	99.9983	99.7911	99.8373	99.9321	99.7121
Kappa comp		99.8393	99.9988	99.9959	99.9984	99.9991	99.9629	99.8866	99.9849	99.7585
Kappa junk		96.0655	99.9961	99.9931	99.9972	99.9992	99.4871	99.8209	99.8755	99.7636
Lambda		99.5378	99.9966	99.9927	99.9986	99.998	99.7235	99.8559	99.9348	99.8478
Lambda comp		99.8652	99.9971	99.9943	99.9987	99.9983	99.9648	99.8958	99.978	99.8645
Lambda junk		97.3685	99.9925	99.9891	99.9984	99.9974	99.2406	99.6707	99.8151	99.7704
Heavy	Accuracy	99.8660	99.9999	99.9998	99.9999	99.9985	99.9928	99.9732	99.9832	99.9925
Heavy comp		99.9744	100	99.9998	99.9999	99.9985	99.9978	99.9771	99.995	99.9943
Heavy junk		96.4897	99.9995	99.9987	99.9997	99.9974	99.8687	99.8316	99.8235	99.9539
Kappa		99.6746	99.999	99.998	99.9979	99.9995	99.8210	99.8919	99.9606	99.8314
Kappa comp		99.897	99.9991	99.9985	99.9987	99.9997	99.9862	99.9372	99.9948	99.8682
Kappa junk		96.5836	99.9983	99.9974	99.9991	99.9998	99.5863	99.875	99.9139	99.8404
Lambda		99.6249	99.998	99.997	99.9996	99.9985	99.7544	99.9028	99.9539	99.9078
Lambda comp		99.9047	99.9982	99.9975	99.9997	99.9987	99.9751	99.9408	99.9887	99.9179
Lambda junk		97.7400	99.9974	99.9943	99.999	99.9994	99.3573	99.7003	99.8697	99.8458

ANARCII-accuracy was able to correctly number the conserved cysteines (23, 104) and aromatics (41 and 118) residues with over 99.99% agreement to ANARCI in both heavy, kappa and lambda chains (Table 1). Agreement of all the positions of CDR regions was lower, with a minimal agreement of 99.97% for heavy, 99.82% for kappa and 99.75% for lambda chains. By filtering the test sets to ensure all target sequences contained conserved cysteines at positions 23 and 104 we increased the CDR delineation agreement to above 99.98%, 99.94% and 99.94% in heavy, kappa and lambda chains (Table 1—row labelled ‘comp’). The addition of random sequence (termed junk) negatively impacted overall agreement (Table 1—row labelled ‘junk ends’); however, this was also related to truncations where assignment of the start or end residue was further complicated by the presence of random residues (some of which may have resembled viable antibody content). In the CDR regions, the agreement values were slightly lower for ANARCII-speed; however, most values were reduced by less than 0.1% on these challenging test sequences (Table 1).

Given that truncations had a negative impact on agreement, we filtered our test sets to remove these sequences and recalculated agreement with ANARCI numbering (Table 2). This boosted CDR1 and CDR3 agreement to 99.99% for all chains using ANARCII-accuracy, while conserved residue agreement was close to 100% (lowest value of 99.9988%, lambda chains with junk). CDR2 agreement was lowest at 99.95% for heavy chains, 99.99% for kappa and 99.97% for lambda chains. Truncated sequences are commonly found in NGS datasets^25^ and the absence of start or end residues creates challenges for both ANARCI (HMM) and ANARCII (language models) and could explain the poor agreement in this area. However, this could not explain the disagreement within the CDR2 and DE-loop^35^ regions, where agreement was worst despite this region being less diverse than the CDR3 region. We next investigated the numbering of sequences, which were inconsistently labelled between different versions of ANARCI to see whether the CDR2 and DE-loop region was also the primary source of ambiguity and whether the use of an additional numbering method could help find a ground truth.

**Table 2:** ANARCII models performance on full length non-truncated test sequences. % Agreement with ANARCI

Test set	Model	Overall	CYS-23	CYS-104	TRP-41	Ar-118	CDR1	CDR2	CDR3	DE-Loop
Heavy	Speed	99.8192	99.9999	99.9998	99.9999	99.9992	99.9963	99.9838	99.9953	99.9907
Heavy comp		99.9544	99.9999	99.9999	99.9999	99.9993	99.9969	99.9834	99.9974	99.9909
Heavy junk		95.9818	99.9988	99.9988	99.9991	99.999	99.979	99.8581	99.9808	99.945
Kappa		99.5819	99.9998	99.9994	99.9998	100	99.996	99.9695	99.9965	99.9361
Kappa comp		99.8389	99.9998	99.9994	99.9998	100	99.9976	99.9669	99.9982	99.9340
Kappa junk		95.8878	99.9996	99.9991	99.9991	99.9998	99.9879	99.9805	99.9928	99.9560
Lambda		99.5134	99.9998	99.9981	99.9996	99.9999	99.9906	99.9454	99.9878	99.9092
Lambda comp		99.8648	99.9999	99.9981	99.9996	99.9999	99.9984	99.9458	99.994	99.9116
Lambda junk		97.2163	99.9995	99.998	99.9995	99.9994	99.9842	99.9495	99.9776	99.8939
Heavy	Accuracy	99.8632	100	100	100	99.9996	99.9987	99.9922	99.9969	99.9962
Heavy comp		99.9744	100	100	100	99.9996	99.9991	99.9919	99.9987	99.9963
Heavy junk		96.4010	99.9996	99.9996	99.9998	99.9996	99.9887	99.9549	99.9875	99.9732
Kappa		99.6645	99.9999	99.9999	99.9998	99.9999	99.9963	99.9902	99.9936	99.9823
Kappa comp		99.8968	99.9998	99.9998	99.9998	99.9998	99.9979	99.9905	99.9991	99.9846
Kappa junk		96.4539	99.9999	99.9998	99.9997	100	99.9953	99.9889	99.9975	99.9743
Lambda		99.6068	99.9998	99.9994	99.9998	100	99.9917	99.9665	99.9934	99.9457
Lambda comp		99.9045	99.9999	99.9995	99.9998	100	99.9986	99.9684	99.9984	99.9494
Lambda junk		97.6322	99.9997	99.9988	99.9997	99.9998	99.9896	99.9725	99.988	99.9357

### ANARCII outperforms existing methods in numbering of challenging sequences

As described in the methods, we identified sequences where the number labels assigned by the latest version of ANARCI diverged from the initial numbering deposited in OAS. These differences could be explained by changes in the germline reference sequences used to construct the HMMs, which underpin ANARCI. For example, the addition of new alleles to the IMGT reference set may result in alternate numbering of certain sequences between versions of ANARCI built with, and without, this updated information. We filtered these sequences to identify those with the greatest difference between ANARCI versions to create a challenging dataset, the ‘no-truth’ set. We numbered the no-truth set sequences with ANARCI, ANARCII-accuracy, ANARCII-speed and a rapid alignment-based tool, AntPack^15^. Given their ease of use and ability to run on mixed sets of sequences from multiple species, we performed a detailed comparison of AntPack^15^ and ANARCI^22^ numberings to ANARCII. We did not compare to other tools, such as IgBLAST^19^ and IMGT/V-Quest^18^ as they both require knowledge of the query species to run. This is not practical for tasks such as rapidly screening large numbers of sequences from many different species, or numbering ambiguous engineered, or novel, formats. In addition, IgBLAST does not perform explicit numbering, only region delineation, and IMGT/V-Quest cannot be run locally and must be used as a web server.

Analysis of numberings in the no-truth set revealed that agreement was highest between ANARCII-speed and ANARCII-accuracy, with 91.1% of sequences numbered identically, and most disagreements falling in the CDR3 region (Supplementary Fig. 3A, B). As ANARCII-accuracy showed marginally higher identification of conserved Cys104 residues (276 more identified, difference of <0.1% across all ~900K no-truth sequences), we focused on comparisons between ANARCII-accuracy, ANARCI and AntPack for the remainder of the analysis.

**Fig. 3:**
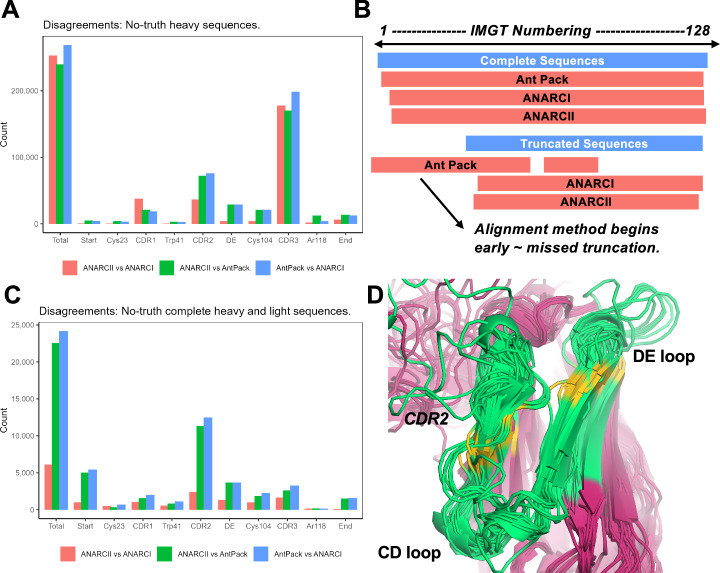
Disagreements between numbering tools and structural variation in the CDR2-DE-loop region. **A** Comparison of AntPack, ANARCI, and ANARCII-accuracy on ‘no-truth’ heavy sequences (where two versions of ANARCI failed to agree) according to the sequence region where the disagreement occurs. **B** Schematic of how numbering tools deal with truncated sequences. **C** Corresponding disagreement analysis of complete heavy and light sequences found in the ‘no-truth’ set where 2 of three methods could identify the residues labelled 2 and 127. **D** Alignment of antibody structures in SAbDab where ANARCI and ANARCII-accuracy disagree in the CDR2 to DE loop region of the heavy chains. IMGT residues 55-90 are coloured green from the beginning of the CDR2 to the end of the DE loop. Residues associated with the loop start/ends (CDR2: 55, 66; DE: 80, 86) are coloured in gold. PDB codes used: 3RPI, 4P9H, 4P9M, 4RWY, 4RX4, 4YDL, 5A7X, 5A8H, 5C0N, 5C7K, 5CJX, 5DMG, 5F21, 5JS9, 5JSA, 5THR, 5TRP, 5V6M, 5VIY, 5VJ6, 6CM3, 6DBD, 6EDU, 6MF7, 6NQD, 6URH, 6XJA, 6XYM, 6XZF, 6Y0E, 7F5H, 7FBI, 7JOO, 7KDE, 7MXE, 7OW1, 7OXN, 7T6X, 7UYL, 7UYM, 7YOY, 8BW0, 8C7J, 8CZZ, 8D50, 8DOK, 8G6U, 8GPI, 8GPJ, 8IM1, 8R4B, 8R4D, 8TTW.

When compared to ANARCI (recent version), ANARCII-accuracy (Fig. 3A) numbered 52.4% of heavy sequences identically. In sequences where numberings were not identical, labels mainly diverged in the CDRH3 in long stretches of sequence containing multiple aromatic, or non-standard residues around position 118 (for example, runs of repeated tyrosine residues from positions 116 to 119). Comparison of numberings between AntPack with ANARCI (49.5% of sequences with identical numbering) and AntPack with ANARCII-accuracy (54.9% identical) were similar; however, AntPack disagreed with ANARCI and ANARCII-accuracy at Cys23 in 0.7% of sequences and Cys104 in 4.0% of sequences. Inspection of these disagreements revealed that AntPack was indicating a non-Cys amino acid at positions-23 and 104, in 0.5% and 4.0% of no-truth heavy sequences, respectively. Of the three methods, ANARCII-accuracy mislabelled the fewest Cys23 and Cys104 residues, with values of less than 0.1%. This exemplified that ANARCII-accuracy was making fewer obvious mistakes than both existing methods when working in highly ambiguous settings.

Further analysis showed most of the affected sequences had a truncation in the first 50 residues. If the truncation occurred before the Cys23, AntPack would begin with a number label close to 1, wrongly assigning the conserved cysteine then ‘catching up’ by placing a large ~10 residue deletion in the CDR1 so that the subsequent sequence was correct (correct identification of Trp41) (Supplementary Fig. 3C). If the sequence was truncated at the CDR2 region (residues 50–60), then AntPack would again number with a label between 1 and 20, incorrectly assigning the entire sequence and placing a large deletion in the CDR3 to allow correct numbering of the aromatic at position 118. We also noted that several sequences where ANARCI and ANARCII-accuracy agreed, but AntPack did not, contained ‘X’ at positions early in the sequence, in place of standard amino acids.

This indicated that a large fraction of discrepancies between methods were a result of heavy sequence truncations or low-quality reads (presence of an ‘X’), which are commonly observed in OAS and NGS data^25^. While this caused disagreements between ANARCI and ANARCII-accuracy, it disproportionately impacted the ability of the alignment-based method (AntPack) to correctly identify the conserved Cys23 and Cys104 positions (Fig. 3B). For light chains in the no-truth data, the proportion of identically numbered sequences between the three methods was much higher. Only 4.3% of sequences were not numbered identically between ANARCII-accuracy and AntPack, and only 1.6% between ANARCII-accuracy and ANARCI. Here, the proportion of mislabelled Cys23 and Cys104 by AntPack was lower (<0.1% and 0.5%), while ANARCII-accuracy was still the most accurate of the three methods (<0.1% Cys23/ Cys104 mislabelled) (Supplementary Fig. 3D).

Filtering for complete heavy and light sequences (where 2 of the 3 methods identified residues labelled 2 and 127) revealed that AntPack was more in line with ANARCI/ANARCII-accuracy (AntPack mislabelled: 0.25%, ANARCII-accuracy mislabelled: 0.1%), and most disagreements now fell within the CDR2 to DE-loop region (Fig. 3C). We inspected the IMGT germline numberings of this region and found that many sequence patterns in the no-truth set diverged substantially and had no clear way of being annotated.

We next identified structures from SAbDab where ANARCI/ANARCII-accuracy labels disagreed in this region to see if there was any structural pattern to aid numbering (Fig. 3D). Our analysis (Supplementary Figs. 4 and 5, and Supplementary Note 2) showed no consistent choice for how to number the CDR2 to DE-loop region. However, we found that both ANARCI and ANARCII-accuracy were consistently able to identify the conserved residues (Cys23 and Cys104) as well as agree on the CDR1 and CDR3 loops. So while the CDR2 to DE-loop region is challenging to number in some sequences, it did not impact the delineation of other CDR loops.

### Improved identification and numbering of rare antibody sequence types by ANARCII

**Fig. 4:**
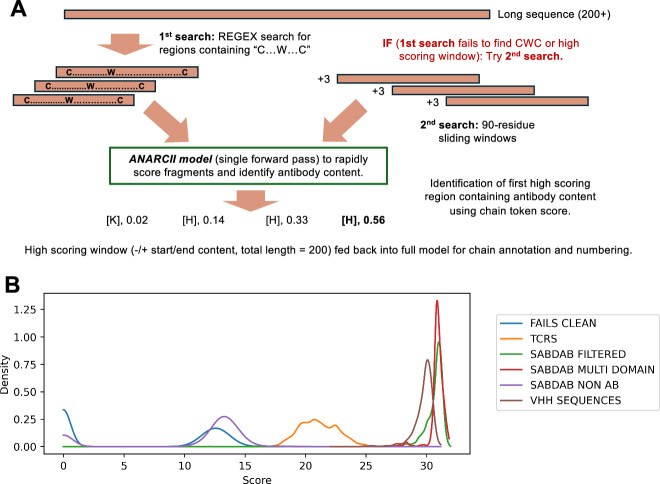
Preprocessing steps allow numbering of long sequences present in the PDB. **A** Sequences longer than 200 amino acids are first screened for a CWC pattern to find chunks which may represent antibody content. The regions with CWC matches are each passed to ANARCII in a single forward pass of the decoder to obtain the corresponding score of the chain token. The first match which scores over a defined score threshold is then fed back into the full model with extra sequence at the start (-40 from the primary cysteine) and end (+160 from the primary cysteine) to ensure complete numbering. If no window passes the threshold or no CWC matches are found, then each sequence is broken up in 90 residue windows which increment every 3 residues. These windows are then scored as before, with the first window over a threshold being run back into the model (-40, window, +70). If no window passes the threshold, then the highest scoring window is chosen. **B** Density plot of sequence scores output by the model (mean of top token scores) for numbered sequences derived from SAbDab, 100,000 TCR sequences, PLAbDab-nano (VHH) and 100,000 non-antibody sequences (Fails) retrieved from UniProt. SAbDab sequences are divided into non-antibody chains (NON AB), multi-domain chains containing more than one FV regions (MULTI DOMAIN) and chains with only on FV region (FILTERED).

An important use case of the predecessor tool, ANARCI, is to identify antibody sequences present in the PDB^34^. This presented a challenge for ANARCII as the PDB contains many long sequences which exceed the length of the context window (210 tokens) and therefore require pre-processing into smaller chunks to permit identification and numbering of the antibody content. To allow the model to process long sequences, we employed a combination of pattern matching and sliding window approaches to break up the sequence into fragments, which are scored with a one-step decoder that identifies the region most likely to contain antibody content (Fig. 4A, see ‘Methods’). The first region predicted to contain antibody content from each long sequence is then passed to the full model for complete numbering. The model then outputs a score for each sequence derived from the sum of the logit values assigned to each integer token (non-insertion).

This method allowed us to process all sequence data from the PDB (downloaded September 2025) and demonstrated that ANARCII-accuracy sequence scores could be used to correctly identify over 99.9% of PDB codes previously identified as antibodies and present in SAbDab^36^. We then examined the distribution of sequence scores from all SAbDab data (divided by single, multi-chain or non-antibody), a set of 100K TCR sequences and a set of VHH sequences (from PLAbDab-nano^33^) and a set of 100K UniProt sequences filtered to exclude immunoglobulins (labelled ‘Fails’ in Fig. 4B). ANARCII-accuracy could discriminate between antibodies and non-antibody proteins and assign high scores to VHH sequences despite these being subtly different from conventional (VH) heavy chain sequences.

However, in our analysis of all sequences in the PDB, we found over 200 PDB codes which contained antibodies and were not present in SAbDab. While most had been discounted due to issues with the structural data (missing residues in the CDRs or lack of electron density), we identified many sequences which were missed due to the inability of ANARCI to label them as antibodies. These sequences/structures predominantly included rarer formats such VNARs and non-hypervariable immunoglobulin domains with very high levels of homology to antibodies (such as CD8A, for details see Supplementary Table 4, for example see PDB codes: **8HT3, 3BDB, 7SPP**). For these antibody-like sequences, the generalisation capacity of ANARCII-accuracy allowed it to find a suitable numbering that resulted in the identification of conserved residues and loops analogous to CDR1 and CDR3. While the numbering applied to these sequences looks highly plausible and may confound efforts which seek to screen mixed sequence sets for antibodies, they had confidence scores which fell outside the distribution of bona fide antibodies found in SAbDab (see Fig. 4B). The ANARCII codebase provides an option to flag such outliers to the user for further inspection. In addition, we provide guidance on changing the score cutoffs to reduce the number of false positives during screening if identification of rare formats is not a priority (https://github.com/oxpig/ANARCII/wiki/Using-ANARCII-as-a-screening-tool).

We have added the VNAR structures to SAbDab (see full list of added PDB codes, Supplementary Table 4). As SAbDab is used as training data for several antibody-specific structure predictors, we expect these new additions, which fall on the fringes of the current structural distribution, to have a direct impact on the next generation of models trained on this data.

Identification and numbering of these unconventional antibodies in the PDB led us to explore in detail how ANARCII-accuracy dealt with sequences from species and types not seen in training data that standard approaches typically fail on.

### ANARCII generalises to unseen sequence types and can be conditioned to create sensible data for fine-tuning

Increasingly, researchers are exploring alternative immunoglobulin modalities to develop novel therapeutic platforms that can address disease challenges^37,38^. An example is VNARs, which utilise shark V-genes and contain sequence features such as large gaps or enrichment of cysteines that diverge from the standard format of normal antibody heavy chains (Supplementary Fig. 6)^39,40^. Tools such as ANARCI and AntPack struggle to number such sequences as their methodology cannot generalise beyond the patterns captured by their reference sets/consensus sequence.

This was exemplified when we examined performance on VHH and VNAR sequences. For VHH sequences, overall agreement between methods was high. AntPack was able to identify the conserved Cys23 residue in three sequences missed by both ANARCI and ANARCII, while ANARCI and ANARCII correctly identified the conserved Cys104 residue in four sequences missed by AntPack. Overall disagreement was low (143 of 4363 sequences), with most disagreement in the CDR2/DE region (56 sequences) or in very long, or truncated, CDR3 regions (19 sequences). Of these latter cases, six were correctly numbered by ANARCII, two by AntPack, and the remainder could not be unambiguously resolved.

Performance on VNAR sequences was poor for both ANARCI and AntPack. ANARCI failed to return a numbering for all 456 VNAR sequences, while AntPack failed to number 99 of 456 sequences. For those sequences successfully numbered by AntPack, predicted CDR3 lengths were shorter (median = 12, mean = 12.9) than those predicted by ANARCII (median = 19, mean = 17.9). Known VNAR CDR3 sequences follow the length distribution shown by ANARCII. This discrepancy suggests misidentification of internal CDR3 cysteine residues as the conserved framework Cys104 by AntPack, and required us to analyse this region using available structural data.

**Fig. 5:**
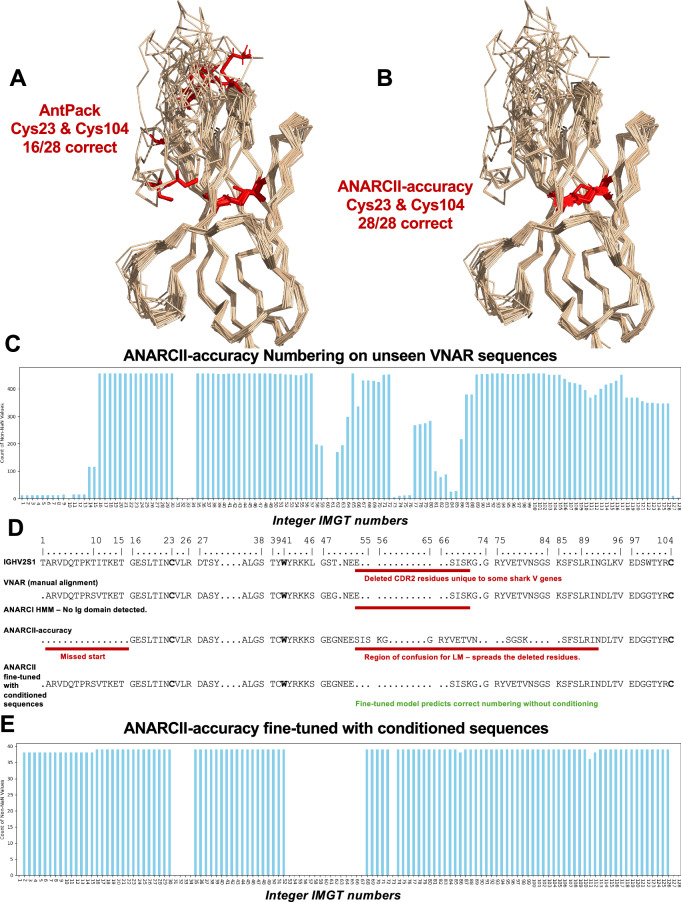
Conditioning and fine-tuning allow numbering of rare sequence types. Highlighting in red the positions identified as the conserved Cys23 and Cys104 in 28 PDB structures of VNARs numbered with AntPack (**A**) or ANARCII-accuracy (**B**). PDB codes are: 1SQ2, 1T6V, 1VER, 1VES, 2COQ, 2I24, 2I25, 2I26, 2I27, 2YWY 2YWZ, 2Z8V, 2Z8W, 3MOQ, 4HGK, 4HGM, 5L8J, 5L8K 5L8L, 6X4G, 6X4T, 7FBJ, 7FBK, 7S83, 7SPO, 7SPP, 8HGI, 8HT3. **C** Bar plot of IMGT number frequencies (insertions not shown) from numbering of 456 VNAR sequences by ANARCII-accuracy. **D** Analysis of a VNAR sequence against the closest corresponding nurse shark IMGT annotated V-gene as well as numbering by ANARCII-accuracy and a model fine-tuned on conditioned sequences. **E** Bar plot of IMGT number frequencies (insertions not shown) from numbering of 37 held out VNAR sequences in the test set by ANARCII-accuracy fine-tuned on conditioned sequences.

Figure 5 shows the numbering of 28 VNAR structures from the PDB by AntPack (Fig. 5A) and ANARCII-accuracy (Fig. 5B). In 12 of 28 sequences numbered by AntPack the Cys104 was misidentified and placed within the CDR3 (Fig. 5A). In ANARCII-accuracy numbering, all the Cys104 residues were placed correctly within the framework, demonstrating the model was able to generalise and produce consistent, structurally sensible numbering at key residues.

We then inspected ANARCII-accuracy numbering of 456 VNAR sequences retrieved from PLAbDab-nano^33^. Conserved residues Cys23, Trp41 and Cys104 were correctly numbered in 97% of sequences (444/456), with the majority of numbering beginning from position-16. However, the CDR2 deletion was not numbered consistently, with gaps being spread across residues 57 to 90 (Fig. 5C, see example sequence in Fig. 5D). In 278 sequences, ANARCII-accuracy identified an aromatic at 118, while 25 were a cysteine, with the remaining dominated by charged residues (E, K, D, H) or unlabelled (model could not confidently number beyond residue 117 for 88 sequences).

Identification of where ANARCII-accuracy was failing to number correctly (by comparison to IMGT germline annotations of a shark V gene, Fig. 5D) allowed us to perform a simple correction at the time of autoregressive inference; this correction is commonly referred to as ‘conditioning’^41^. To do this, we identified patterns associated with IMGT labels and overwrote the predicted token at the inference step. The corrected token was then passed to the decoder to continue numbering (Supplementary Note 3). Through application of these conditioning steps, we were able to rapidly rerun the numbering of large sets of shark sequences and obtain numbered outputs which fully aligned with IMGT germline definitions.

We then fine-tuned ANARCII-accuracy on these outputs to obtain a model that could correctly identify the CDR2 gap, GXGT motif and conserved residues in all cases, without any conditioning steps (Fig. 5E). This test case demonstrates the ease of conditioning and fine-tuning to build on the fundamental sequence patterns learnt by ANARCII-accuracy. We have included this fine-tuned model, ‘VNAR-accuracy’, in the ANARCII package and webapp to allow users to explore its predictions and accurately number VNAR sequences, as well as conditioning code and instructions for customisation.

The conditioned outputs formed the basis of the first comparative analysis between VNARs and VHHs sequences presented in Gordon et al.^33^ and exemplified a fundamental use case of ANARCII in an exploratory setting. As new antibody species data and receptor formats are analysed, we anticipate ANARCII will be increasingly used in this way.

### ANARCII models are capable of numbering large portions of TCR sequences correctly, and achieve high accuracy with minimal fine-tuning

**Fig. 6:**
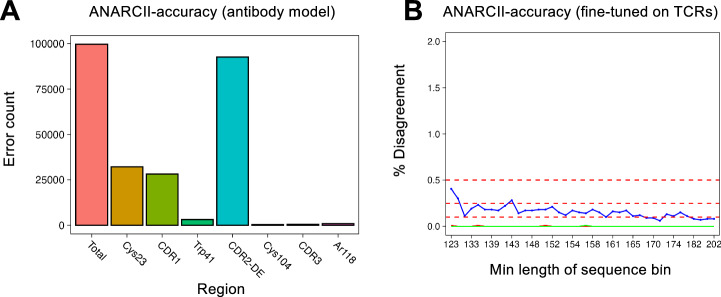
ANARCII-accuracy can identify conserved residues and CDR3 loops of unseen TCR sequences and undergo fine-tuning for complete identification. Numbering errors from ANARCII-accuracy labels on 100K TCR sequences (**A**) binned by region and conserved residues. Disagreement in numbering of ANARCI and TCR-accuracy model versus sequence length on a held-out test sets of 0.4M TCR sequences (**B**). Test sequences are divided into 40 bins based on length, indicated on the *x* axes, from short to longer sequences.

Both ANARCII-accuracy and ANARCII-speed models were able to number TCR sequences in a similar manner to VNARs (correct identification of conserved residues, divergence around the CDR2/DE region, Fig. 6A); however, their chain token assignments were incompatible. For comparison, providing a TCR sequence to ANARCI and restricting the model to HMMs built from antibody germlines fails to return any alignment. We were able to expand the weights to accommodate the four TCR chain types (alpha, beta, gamma, delta) and then rapidly fine-tune both models on TCR data, resulting in high-accuracy versions with minimal training (Fig. 6B). We have provided options to run the TCR-accuracy and TCR-speed models within the ANARCII package and webapp.

### Alternate numbering schemes can be obtained through conversion of IMGT outputs

To number in alternate numbering schemes such as Kabat^7^, Martin^9^ or Aho^10^ we have modified the methodology used by ANARCI^22^. Instead of converting state vectors from HMM outputs, the conversion algorithm processes the final numbered outputs from an ANARCII model and returns the updated scheme. Once a sequence, or set of sequences, has been IMGT numbered, the user can rapidly and repeatedly convert to Kabat, Martin, Chothia or Aho without having to rerun the numbering step (see user guide at https://github.com/oxpig/ANARCII/wiki). In addition, the web app provides functionality that allows users to inspect IMGT numbering alongside an alternate scheme to visualise how the new numbering diverges from IMGT labels (https://opig.stats.ox.ac.uk/webapps/sabdab-sabpred/sabpred/anarcii/).

We have also implemented optional flags in the ANARCII codebase that return warnings when sequences fall in a low confidence zone and thus warrant further inspection. An additional flag allows for raw logits to be returned for all positions in a numbered sequence, allowing users to inspect of relative scoring of positions and regions (see https://github.com/oxpig/ANARCII/wiki/Model-arguments-and-methods). Finally, for users with complex pipelines or codebases built on ANARCI, we have provided a legacy function which can be used to convert the ANARCII outputs to the original ANARCI format to minimise disruption.

## Discussion

Computational numbering of immune receptor sequences was once a simple task that is becoming increasingly complex due to growing quantities of sequencing data from a diversity of species, alleles and receptor formats. Previously successful methodologies based on aligning to a set of reference sequences are now limited in their ability to accurately number rare sequences that may adopt unusual structural patterns. As the reference sets change and grow, tools such as ANARCI exhibit inconsistencies in how they number the same sequences and are ultimately reliant on downloading from an external database at the time of installation. Furthermore, alignment-based tools struggle when numbering truncated sequences that are very common in NGS datasets.

We have designed ANARCII to address these challenges. By training a small language model architecture to carry out the task of antibody sequence numbering, we developed a tool that is comparable to standard approaches, whilst relying only on its internal weights and not requiring the external download of evolving reference sets. In addition, we observed that for challenging sequences, ANARCII exhibited better identification of key conserved residues and was more robust to truncations than existing approaches. Importantly, the model was able to generalise to number rare VNAR sequences where standard approaches failed to return an alignment. The flexibility of autoregressive inference allows for model labels to be modified or ‘conditioned’ to fit arbitrary sequence patterns. Using this method of conditioning, we were able to create data used in the first comparative analysis of VNAR and VHH sequences without manual annotation or multiple sequence alignment.

We anticipate that the ability of ANARCII to generalise to lesser-known areas of the antibody sequence space will facilitate further analyses and annotation of new species and receptor formats without relying on manual annotation or the generation of IMGT reference sets. Some further examples are the model’s ability to number non-antibody immunoglobulin sequences such as CD8, PDL1, SIRPa and TREM2, which are present on the surface of immune cells and bind to conserved targets using loops analogous to CDRs. Some of these proteins have recently been used as training data for antibody-specific deep learning models^42^; however, like VNARs, they cannot be numbered with existing tools and serve as another example of where ANARCII can bridge the gap to other members of the antigen-receptor family.

An additional advantage of ANARCII is the improved intra-sequence recovery, specifically the numbering of long CDRH3 sequences in cases where existing methods stop prematurely. For example, we renumbered sequences in paired OAS with ANARCII and found over 2000 chains with long CDRH3 sequences (out of 3M, 0.07%), where ANARCI had failed to identify the true CDRH3 end, and incorrectly caused a warning (OAS-specific annotation) that the CDRH3 was truncated. While this represents a very small improvement relative to the scale of OAS paired, deep learning models benefit from more data, and particularly points at the edges of the distribution. Antibodies with long CDRH3s fall into this category.

A final advantage specific to ANARCII over ANARCI relates to the installation and compilation of the HMMs. As stated in the introduction, when a user first installs ANARCI they are required to download IMGT reference sets and compile HMM models. This means that as the reference sets change over time, small differences appear in the way ANARCI numbers the same sequence, with numbering outputs for one user differing from another with more a recent compilation. In addition to this being a source of inconsistency, it adds a non-Python dependency to the installation of ANARCI. When the IMGT website is temporarily unavailable (for example, due to routine maintenance or server issues; see GitHub issue #97), users are unable to install ANARCI from source and must instead rely on historic builds, which may diverge from more recent versions. The use of a static language model streamlines the install process and ensures the numbering is consistent for all users.

The primary disadvantage of using a language model instead of simpler alignment methods is the imbalance in computational cost and speed. While the speeds of ANARCII are comparable to ANARCI when users have access to a high-end GPU, running on CPUs is much cheaper, and both methods still lag far behind AntPack (reported to be up to ~60× faster^15^). These differences relate to design choice, ANARCII for generalisation and robustness, AntPack for speed and computational efficiency. If a user is prioritising robustness to truncated sequences or performance on rare sequence types, then ANARCII offers the better choice. If their sequences are highly generic and in such quantities that poorer recovery on truncations is acceptable, then AntPack may be more suitable. In line with the setting-specific advantages of AntPack, if a user is working with well-studied organisms for which receptor germline sequences are available, then IgBLAST and IMGT/V-quest offer excellent alternatives, albeit with limitations in output format and the ability to run locally, respectively.

ANARCII does require a top-end GPU to attain its maximum speeds; however, advances in speed, energy-efficiency and reduction in GPU hardware costs mean that each year running ANARCII is likely to become cheaper and faster^43^. Indeed, recent publications have demonstrated this in settings such as homology search^44^ and long read alignment^45^, where GPU acceleration can improve upon multi-CPU timing benchmarks.

In the biological space, replacing ‘simpler’ methodologies with ML/AI approaches will raise both technical challenges as well as debate and discussion on the acceptance of results derived from black box models. We hope ANARCII exemplifies that thorough analysis of model outputs can impart confidence in these predictions and reveal the many advantages in adopting the latest statistical techniques to solve key informatics problems.

## Methods

### Retrieval of non-redundant heavy and light chain sequences from the Observed Antibody Space

All antibody sequences used were retrieved from the OAS^25,26^. Due to the scale of data in OAS, all files were each randomly sampled to build a representative, non-redundant set of sequences. For heavy chains, a maximum of 100,000 non-redundant sequences with dataset redundancy (the number of reads detected per sequence) greater than 1 were randomly sampled from each file. To compensate for the lower quantity of light chain data, a maximum of 500,000 non-redundant sequences were randomly sampled per file, with dataset redundancy of 1 allowed. Samples were combined and duplicate sequences removed. The number of sequences collected after this step was: ~95.6M heavy and ~75.0M light chains. All sequences were renumbered using ANARCI (Dunbar and Deane 2016) compiled with the latest available germline datasets at the time of analysis (December 2023). Sequences exhibiting clear mislabelling of conserved cysteine positions (IMGT positions 23 or 104) were filtered, identified by the presence of a cysteine immediately adjacent to a non-cysteine residue at a position expected to be strictly conserved. This issue, a known limitation of ANARCI, affected fewer than 0.06% of sequences. This process used all available data in 99.1% of OAS light chain files; however, it left many unsampled heavy sequences (4.0% of heavy files had over 100,000 sequences at redundancy greater than 1, and therefore contained sequences which had not been sampled in the first pass). These unsampled sequences were then used to increase the number of rare insertions and non-human or mouse species contained in the dataset.

### Enrichment of heavy chain sequences for rare insertions and species

We analysed the numbering of all sequences in the heavy dataset and compiled statistics on the number of times each insertion was seen, for example, 112A was seen over 57,448,967 times, whereas 12A was seen only 39 times. We identified all insertion codes that were seen less than 1000 times and resampled all files in OAS that were not fully sampled by the initial processing steps to find sequences with these rarer insertions and added these to the heavy dataset. After this step, we enriched for non-human and non-mouse species; these included rat, rabbit, camel and rhesus macaques. Files from these species, which were partially sampled from previous steps, were further sampled to search for any sequences not already present in the heavy dataset. These steps added 123,305 sequences to the dataset and resulted in a final number of ~95.7M heavy chains. The total number of unique number labels rose from 925 pre-enrichment to 968 post-enrichment.

### Sequence clustering and dataset splits

The levels of dataset diversity in the light and heavy chains are very different; for example, only 143,563 light CDR3 sequences contained insertions at position 112A (the first CDR3 germline insertion position in IMGT numbering) in the ~75.0M sequences, compared to ~57.1M in ~95.7M for the heavy chain. As a result of this, we used different parameters for light and heavy sequence clustering to create large numbers of diverse clusters that could be split into train, test and validation sets. We performed small-scale experiments to identify clustering thresholds that best balanced diversity across train/validation/test splits, minimised sharing of highly similar sequences between splits, and computed. These analyses are described in detail in the supplementary information (Supplementary Note 1, Supplementary Fig. 2 and Supplementary Table 1). Using CD-HIT (Li and Godzik 2006), heavy sequences were clustered at 75% identity, while light sequences (kappa and lambda separated) were clustered at 85% identity across the entire sequence.

Coverage settings were employed to ensure that truncated sequences were clustered with their likely full-length parent sequences. This was achieved by requiring high coverage of the shorter sequence using the CD-HIT aS parameter (set to 0.8 for heavy chains and 0.9 for light chains), while leaving coverage of the longer sequence at the default (aL=0). This configuration allows high-fidelity alignment of truncated sequences without artificially fragmenting clusters due to differences in sequence length. This resulted in 1,067,916 heavy clusters, 1,246,933 lambda clusters and 1,837,998 kappa clusters, which were each split 90:5:5 into train, validation and test sets. The final numbers of clusters and sequences in each set are shown in Supplementary Table 2.

### TCR dataset generation

Paired TCR sequences were extracted from the Observed TCR Space^46^, and unpaired sequences from iReceptor^47^ (for details see Raybould et al. 2024; raw data can be found at 10.5281/zenodo.11208211). Paired sequences were split into individual chains, combined with unpaired data and numbered with the latest version of ANARCI. As ANARCI can falsely identify a certain subset of alpha chains sequences with TRAV/DV genes as delta chains, we utilised IgBLAST^19^ gene calls where available. Using this process, 435 chains in the training data were delta chains. In total, 4,993,877 alpha, 6,067,942 beta and 526 gamma chains were collected for training TCR-specific models.

### Addition of junk and tokenisation of training data

For each sequence in the train and validation datasets, between 0 and 40 random amino acids were added either side of the antibody content to mimic natural non-antibody content often found in antibody sequence datasets and crystal structures. The corresponding target labels of these randomly added residues were defined by a [<SKIP>] token. Starting junk residues were added after the [<SOS>] token and before true antibody content began. After the antibody content ended, an [<EOS>] token was added, followed by random junk residues. The purpose of this was to provide an easy way to identify the end of the sequence during post-inference processing of predicted tokens. The amino acid tokeniser also includes a token for non-standard residues (e.g. B, O, J, U, X, Z) [X] which may represent unknown residues in sequencing methods like NGS or mass spectrometry. The numbering tokeniser used to create target labels included all numbers from 1 to 128, as well as an [X] to mark an insertion. All insertion labels were collapsed to [X] and are translated to the correct letter codes in post-processing. The input and output tokens pass through different embedding layers in the encoder and decoder, respectively; therefore, shared tokens like [X], [H], [L], [K] each have separate learned representations within the source and target sequences.

### Model training

Due to the large amount of training data (~140M sequences, comprised of ~70.5M heavy, ~38.7M kappa and ~29.9M lambda chains), heavy and light sequences were each split into 5 subsets, of which ~10M heavy sequences, ~6M kappa sequences and ~4M lambda were randomly sampled and passed to a dataloader. Validation loss was calculated after all 5 heavy and 5 kappa/lambda subsets had been randomly sampled (in random order), meaning that the model had seen exactly 50M heavy and 50M light datapoints per epoch by our definition. Validation loss was calculated separately for heavy and light sets, then the average validation loss across both chains used for final model selection. A batch size of 512 was found to provide optimal validation loss during testing. Model design and training were performed in PyTorch version 2.2.1^48^, CUDA version 12.1 and Python version 3.11. Model validation and testing were performed in multiple versions of Python (3.9-3.13), PyTorch (2.0-2.5) and Cuda (11.8, 12.1, 12.4) to ensure performance was consistent, and run parameters and calls were all functional across platforms when called from the final package.

### Model architecture and hyperparameter selection

We utilised the standard Seq2Seq architecture described by Vaswani et al.^32^. A set of model hyperparameters was chosen based on convergence over 15-30 epochs (full details shown in Supplementary Table 3, along with corresponding inference speeds on 10,000 sequences). Several models were carried forward to longer training cycles, with the balance of validation accuracy versus inference speed being used to settle on two sets of hyperparameters: a 1-layer model (encoder/decoder hidden dims of 128) with slight reduction in accuracy but faster inference speed (ANARCII-speed), and a 2-layer model (also 128 hidden dims, Supplementary Fig. 1) which was slower but had higher accuracy (ANARCII-accuracy). Ever larger models did give marginal increases in accuracy but also trebled the inference speed relative to the smallest model (Supplementary Table 3).

### Training regime

Three training regimes were tested: a standard step decay learning rate, cosine decay with warm restarts and a warmup phase with cosine decay. Cosine decay with warm restarts every 15 epochs (initial learning rate = 0.0002, minimum = 0.00001) resulted in models with the best validation loss. Each model (ANARCII-speed and ANARCII-accuracy) was trained in this way on an Nvidia-A100-GPU for 75 epochs, then trained for a further 60 epochs with initial and minimum learning rates reduced by a factor of 10 and restarts every 10 epochs.

### Processing of long sequences

The ANARCII models utilised an encoder with a maximum input length of 210 tokens, which therefore could not process very long sequences, which are often found in the PDB (Berman et al.^34^). To permit ANARCII models to number the antibody content of longer sequences, we utilised two strategies to find the relevant 200 residue region, which could be passed to the model to output full numbering. Firstly, for each sequence over 200 residues, we perform a compiled regex search for instances of C, W, C separated by 5-25 and 50-80 residues, respectively. For every match, we pass the content between the two cysteines to the ANARCII model and perform a rapid, single forward pass through the decoder to obtain a score per match derived from the logit value of the predicted chain token (analogous to a <CLS> token). The first high-scoring match is selected, and upstream/downstream sequence added from the beginning cysteine (−40, +160), to give a final sequence length of 200, which is fed back into the full model for complete numbering. If no CWC matches are found, or no high-scoring match is found that exceeds a defined threshold, then a second strategy is employed. Here, the long sequence is broken up into sliding windows of 90 residues (along an increment of 3 residues), and each window run through ANARCII in a single forward pass of the decoder. The first high-scoring window, including flanking sequence (−40 from start, +70 from end, giving 200 residues), is then passed into the full model for complete numbering.

### Sequence classifier for unknown sequences

A classifier was developed to facilitate a two-step ‘unknown’ mode where input sequences are first classified as either antibody or TCR, and then passed to the correct numbering model. The classifier model architecture was identical to the ANARCII-speed model (1-layer, 4 heads and 128 hidden dims in the encoder and decoder), only differing in the decoder output where a single chain token was predicted (<TCR> or <Ig>). The model was trained using sequences sampled from the train, validation and test datasets generated previously (see sections ‘Sequence clustering and data splits’ and ‘TCR dataset generation’). For the antibody data: heavy, kappa and lambda sequences were each separately sampled to obtain an even distribution across the clusters found using CDHit^49^. This resulted in ~3.9M heavy, ~4.2M kappa and ~4.7M lambda training sequences, with ~0.3–0.8M sequences in the corresponding test and validation sets. All TCR sequences from train, validation and test sets were used, resulting in ~4.4M alpha and ~5.5M beta training sequences with ~0.3–0.4M sequences in the corresponding test and validation sets (gamma and delta sequences were highly underrepresented as previously described). The model was trained for 80 epochs using cosine decay (learning rate parameters identical to the initial stage of training for ANARCII-speed, batch size of 256), before reaching 100% accuracy on the validation set.

### Webapp and package

The suite of ANARCII language models can be selected and run from a webapp (https://opig.stats.ox.ac.uk/webapps/sabdab-sabpred/sabpred/anarcii), where output files can be downloaded in CSV or MessagePack (msgpack) format. Users can visualise the differences between IMGT numbering and the alternate scheme requested through a side-by-side sequence analysis tool. The suite is also available to download (https://github.com/oxpig/ANARCII).

### Screening of PDB sequences

To evaluate the performance of ANARCII on real-world structural data, we applied it to the Protein Data Bank (PDB) and assessed agreement with SAbDab annotations. An up-to-date PDB seqres file was downloaded on a fixed date (14-Sept-2025). In parallel, all antibody information, including PDB entry and chain annotations was downloaded from SAbDab (https://opig.stats.ox.ac.uk/webapps/sabdab-sabpred/sabdab) at the same timepoint and used as a reference set.

All sequences contained in the PDB were processed using ANARCII-accuracy run in antibody mode on an A100 GPU. Sequence chain labels (H, L, K or F), numberings and confidence scores were inspected. Recovery of PDB codes and chains was assessed against their corresponding SAbDab labels of antibody, multi-chain antibody and non-antibody content. Recovery was used to inform the default ANARCII-accuracy cutoff score (15) as well as identify a more stringent score range (22–25) that reduced false positives but was not suitable for highly novel receptor types and rare formats (find guidance in the Wiki: https://github.com/oxpig/ANARCII/wiki/Using-ANARCII-as-a-screening-tool). Additionally, we ran ANARCII-accuracy on a set of 100K UniProt non-antibody sequences (length between 90 and 200, excluding keywords related to immunoglobulin), VNAR and VHH sequences from PLAbDab-nano^33^ and TCR sequences from STCRDab^50^. The distribution of sequence scores was assessed for each ANARCII model (only ANARCII-accuracy shown in the final manuscript) and plotted to exemplify the discriminative capacity across different sequence types.

### Creation of the ‘no-truth’ dataset

All data collected for antibody heavy and light chains were retrieved from OAS with the ANARCI numbering created at the time at which the datasets were first processed (between 2018 and 2022). Each sequence was also renumbered with a more recent version of ANARCI (compiled December 2023) and the two numbering versions compared. This highlighted ~3.6M heavy (3.8% of total heavy) and ~0.9M light sequences (1.2% of total light) where there was some disagreement between ANARCI versions. We filtered these to find sequences with large variation in number labels (over 4 different number labels between the two versions), resulting in 530,804 heavy and 410,148 light sequences that we refer to henceforth as the ‘no-truth’ dataset.

### Conditioning of outputs for numbering of VNAR sequences

ANARCII-accuracy was used to number 456 shark VNAR sequences retrieved from PLAbDab-nano^33^. These numberings were compared to the IMGT germline reference for nurse shark V-genes (those with a large CDR2 deletion e.g. IGHV2S1) to understand where the language model predictions differed. The autoregressive inference step was modified to search for sequence patterns and predicted numbering (using regular expressions) that corresponded to where the predictions differed from the germline reference set. When one of these patterns was detected, the number labels were changed to exactly match the IMGT reference labels. The corrected numbering was then fed back into the decoder to continue next token prediction of number labels for the remaining sequence until completion. A detailed description of the sequence patterns and corresponding numbering can be found in the supplementary information (Supplementary Note 3). A Jupyter notebook containing all code and instructions on how to perform and customise the conditioning of number labels can be found at: https://github.com/oxpig/ANARCII/blob/main/notebook/conditioning.ipynb.

### Fine-tuning on shark VNAR sequences numbered by conditioning

VNAR sequences, which had been numbered through conditioning of ANARCII-accuracy outputs (see previous section), were filtered to ensure correct identification of conserved residues (Cys23, Cys104 and Trp41) and the GXGT motif marking the end of the CDR3 (397 out of 456). These 397 sequences were randomly split 80:10:10 into train, validation and test sets. Datasets were upsampled by a factor of 10 (to ensure that the same sequence was present multiple times with different junk context), then randomly shuffled and a junk sequence added (see previous section, ‘Addition of junk sequence and tokenisation of training data’). The ANARCII-accuracy model was fine-tuned on these data for 80 epochs at a reduced batch size of 256 (warm restarts every 10 epochs, initial learning rate = 0.0002, minimum = 0.00001).

### Fine-tuning on TCR data

TCR sequences (see TCR dataset generation section) were clustered using CD-Hit at the level of 85% identity across the entire sequence and split 90:5:5 into train, test and validation sets. Sequences were numbered with the most recent version of ANARCI (compiled December 2023). Junk sequence was randomly added to the start and end of each sequence as was carried out for antibody sequences (see previous section, ‘Addition of junk sequence and tokenisation of training data’). Fine-tuning was performed on both antibody-specific models (ANARCII-accuracy and ANARCII-speed) with weights expanded to accommodate the TCR-specific tokens for chain call (A, B, D, G). Models were fine-tuned for 80 epochs using identical learning rate parameters to the second stage of training at a reduced batch size of 256 (warm restarts every 10 epochs, initial learning rate = 0.0002, minimum = 0.00001).

### Statistics and reproducibility

No inferential statistical tests were performed. All reported performance results are descriptive and are presented as exact agreement rates, conserved-residue recovery, CDR boundary agreement, or inference throughput, as appropriate. Unless otherwise stated, we refer to the number of sequences in the relevant benchmark set. For structural screening analyses, we refer to the number of PDB chains or PDB entries evaluated. For benchmarking, the unit of analysis was the individual sequence. Train/validation/test splits were created by clustering sequences prior to splitting (90:5:5) to reduce leakage between sets, and all final performance metrics were computed on fixed held-out test sets. All analyses were implemented in Python 3.11 and PyTorch 2.2.1 with CUDA 12.1, and model validation and testing were checked across Python 3.9–3.13, PyTorch 2.0–2.5, and CUDA 11.8, 12.1, and 12.4 to ensure consistent behaviour across software environments.

### Reporting summary

Further information on research design is available in the Nature Portfolio Reporting Summary linked to this article.

## Supplementary information

Supplementary Information

Description of Additional Supplementary Files

Supplementary data

Reporting Summary

## Data Availability

Raw antibody sequences are available from the Observed Antibody Space (OAS) website (https://opig.stats.ox.ac.uk/webapps/oas). Test, train and validation data splits have been deposited in Zenodo (heavy chain: 10.5281/zenodo.19449779; light chain: 10.5281/zenodo.19449805). Raw data used to generate graphs is available in supplementary data file. Score distribution data is available from Zenodo (10.5281/zenodo.19449919). All T cell receptor sequencing data is available from the Observed TCR Space (OTS) website (https://opig.stats.ox.ac.uk/webapps/ots) and unpaired data available from Zenodo (https://zenodo.org/records/11208211). VNAR and VHH datasets used for comparison and fine tuning can be downloaded from the PLABdab-Nano website (https://opig.stats.ox.ac.uk/webapps/plabdab-nano/).

## Code availability

Code is available from GitHub at https://github.com/oxpig/ANARCII. DOI-minted changes and releases are available at Zenodo (10.5281/zenodo.15274840). The tool is also available as a web server for use with a limited number of sequences (https://opig.stats.ox.ac.uk/webapps/sabdab-sabpred/sabpred/anarcii/). For larger datasets we recommend local installation from GitHub (https://github.com/oxpig/ANARCII) or PyPI (https://pypi.org/project/anarcii/).
