# Supplementary material for: ANARCII enables alignment-free antigen receptor numbering using a generalised language model
Source: Commun Biol. 2026 May 21;9:1085. doi: 10.1038/s42003-026-10186-z (PMC13462589; doi:10.1038/s42003-026-10186-z)
Supplement: Supplementary file 1 [file 42003_2026_10186_MOESM1_ESM.pdf]

SUPPLEMENTARY INFORMATION

ANARCII enables alignment-free antigen receptor numbering using a generalised language model

Alexander Greenshields-Watson*, Parth Agarwal*, Sarah A. Robinson*, Benjamin Heathcote Williams, Gemma L. Gordon, Henriette L. Capel, Yushi Li, Fabian C. Spoendlin, Broncio Aguilar-Sanjuan, Fergus Boyles, Charlotte M. Deane

Oxford Protein Informatics Group, Department of Statistics, University of Oxford, 24-29 St Giles', Oxford, OX1 3LB United Kingdom

*These authors contributed equally to this work.

Email: deane@stats.ox.ac.uk

SUPPLEMENTARY NOTES

Supplementary Note 1: Identification of appropriate clustering thresholds which balance training split diversity, difficulty and compute. We undertook a series of small-scale experiments on 7.7M heavy chain sequences (~10% of heavy chain data) to determine the optimal clustering threshold when clustering sequences with CD-HIT[1]. Identity thresholds ranging from 50% to 95% were evaluated in 5% increments. For each threshold, we quantified the number of clusters, the proportion of singleton clusters, cluster size distributions, and computational cost (**see Supplementary Table 1**). To directly assess split stability and generalisation difficulty, we performed 42 independent random resampling of clusters at each identity threshold into a 90% training and 10% combined test/validation split. For each split, we measured the percentage overlap of exact CDRH3 sequences between training and test/validation sets as a proxy for shared similar sequences (**Supplementary Figure 2A**), and the percentage overlap of starting framework (FWH1) sequences as a proxy for balanced germline coverage (**Supplementary Figure 2B**).

Lower identity thresholds (≤70%) generated smaller numbers of very large clusters. When these clusters were randomly assigned to train and test/validation splits they exhibited wide, long-tailed distributions in exact CDRH3 and exact FWH1 sharing. Higher identity thresholds (≥80%) increased the proportion of singleton clusters (>50%), substantially increased compute time, and reduced the difficulty of test and validation splits due to the presence of many near-identical sequences. The distributions of CDRH3 and FWH1 sharing narrowed at 75% identity, where CDRH3 overlap consistently remained below 5% and FWH1 sharing did not fall below 2.5% (median ~10%).

Based on these analyses, 75% sequence identity was selected as the optimal threshold for heavy chains, representing a balance between split stability, sufficient separation to assess generalisation, and compute time. Applying this threshold to the full heavy-chain dataset yielded approximately one million clusters from ~96 million sequences.

Applying the same threshold to light chains resulted in substantially fewer clusters (<200,000 from ~75 million sequences), reflecting lower sequence diversity, fewer insertions, and uneven representation of light-chain germlines in available datasets. To ensure balanced germline coverage, light chains were therefore first separated into kappa and lambda subsets and clustered independently at 85% identity with higher alignment coverage. This produced approximately 1.8 million kappa and 1.2 million lambda clusters.

Supplementary Note 2: Structural exploration of the CDR2 to DE-loop region in sequences where numberings differ between ANARCII and ANARCI. To systematically investigate how numbering aligned with secondary structural features such as loops or beta sheets, we used DSSP[2] to analyse all SAbDab structures numbered with either ANARCI (**Supplementary Figure 5A**) or *ANARCII-accuracy* (**Supplementary Figure 5B**). This revealed a distinct secondary structure pattern alternating between extended strands (coloured pink) and turns/bends (yellow/sand) that were synchronised with IMGT

numbering. We isolated 113 SAbDab chains (57 PDB codes) in which ANARCI and *ANARCII-accuracy* disagreed at the CDR2 to DE-loop (but agreed on the preceding conserved Cys23 and Trp41 residues). Visualising the IMGT numbering-to-structure relationship with DSSP revealed a complex pattern with many insertions, underlining the ambiguity in numbering this region (**Supplementary Figure 5C-D**). This could also be seen structurally from alignment and inspection of the 3D coordinates of the heavy chains (**Figure 3D**) with a large variation in the length and conformation of the DE loop, the distal CD loop and small variations in the bridging framework regions. For both tools, numbering mostly aligned with structural features, with differences arising in placement of insertions and deletions after the CDR2 and either side of the DE-loop (**Supplementary Figure 5C-D**).

Supplementary Note 3: Conditioning steps used to number VNAR sequences. Through inspection of *ANARCII-accuracy* numbering on VNAR sequences from PLabDab-Nano[3] we identified key patterns which preceded 'misnumbering' by the language model (according to IMGT germline annotations of a shark V gene, **Figure 6D**). These patterns were used to perform targeted intervention in the autoregressive inference loop and overwrite the predicted token with the correct token, and then pass the partial sequence into the decoder to continue the inference loop.

- To correct the misidentification of the starting sequence we identified the beginning pattern being missed and labelled the first residue according to IMGT numbering (often 1 or 2).
- Next, we used regular expressions to identify the sequence associated with the deleted CDRH2 region (often flanked by NEE) and labelled the subsequent sequence starting from residue 68 to preserve the large deletion characteristic of VNARs.
- Finally, the CDRH3 was completed (often the model would stop numbering long CDRH3s) by identifying the conserved cysteine at position 104 and the terminal GXGT motif at positions 119-122. Once these two patterns were found then the intermediate sequence was renumbered according to standard rules. Through application of these conditioning steps, we were able to rapidly rerun the numbering of large sets of shark sequences and obtain "correctly" numbered outputs.

Finally, we took the conditioned sequences and filtered to obtain those with conserved cysteines at 23 and 104, tryptophan at position 41 as well as the presence of a GXGT motif at positions 119-121 resulting in 397 complete sequences. We split this dataset (80:10:10) and used it to rapidly fine tune the *ANARCII-accuracy* model. When challenged with 38 sequences from the held-out test set, the fine-tuned model was able to correctly identify the CDRH2 gap and GXGT motif and conserved residues in all cases, and the starting number in 37 of 38 sequence (**Figure 6E**).

SUPPLEMENTARY FIGURES

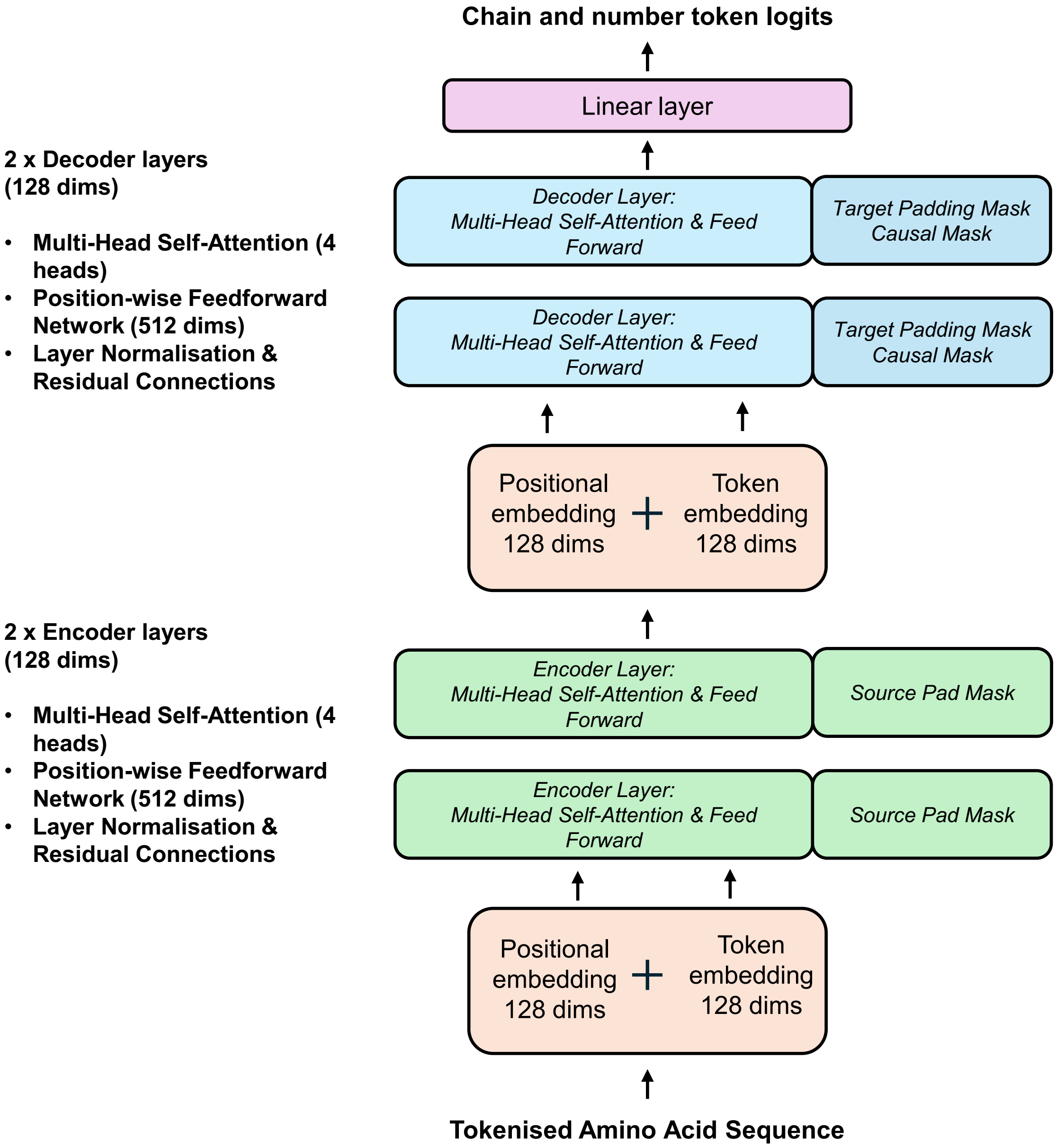

Supplementary Figure 1: Model architecture of ANARCII-accuracy. *Schematic detailing the Seq2seq architecture of ANARCI—accuracy (ANARCII-speed has identical dimensions with only 1 encoder and 1 decoder layer). Blocks indicate the components of the encoder-decoder transformer model used to assign chain and number token logits to input amino acid sequences. The input is a tokenised amino acid sequence (tokens include 20 standard residues, nonstandard residues: X, delimiters: SOS, EOS, PAD), which is first embedded using token and positional embeddings (128 dimensions each), summed and scaled. This representation is passed through 2 encoder layers (128 dimensions, 4 attention heads), each consisting of multi-head self-attention, position-wise feedforward networks (512 dimensions), layer normalisation, and residual connections. The encoded input sequence is then passed to the decoder, which processes the representation using the same architecture (2 decoder layers, 128 dimensions, 4 attention heads) and*

additional causal and padding masks. The final decoder output is projected through a linear layer to 136-dimensional logits, corresponding to chain (H,K,L), insertion (X), delimiter (SOS, EOS, SKIP, PAD) and number label (1-128) tokens.

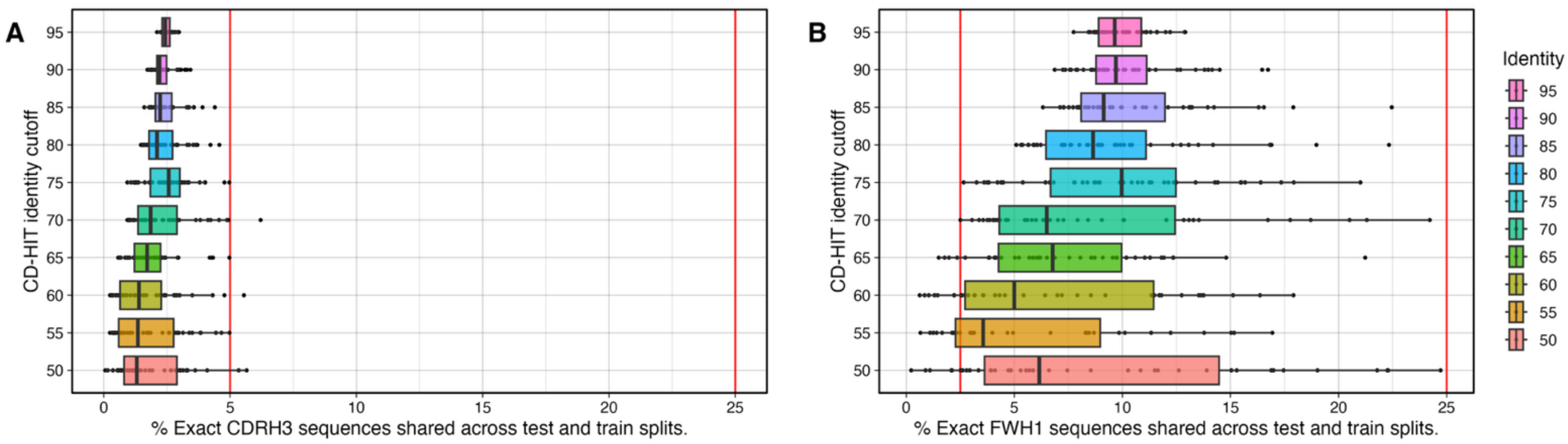

Supplementary Figure 2: Identifying appropriate CD-HIT thresholds to generate data splits. *Results of random resampling over 42 seeds of CD-HIT clusters into a 90% train and 10% combined test and validation split. Overlap was calculated for each seed as percentage of sequences where the exact CDRH3 (**A**) or starting framework (FWH1, **B**) was found in both the 10% fraction and the 90% training set. Each data point represents 1 seed (n=42) and box plots show median and interquartile range (IQR) with whisker length equal to 1.5*IQR.*

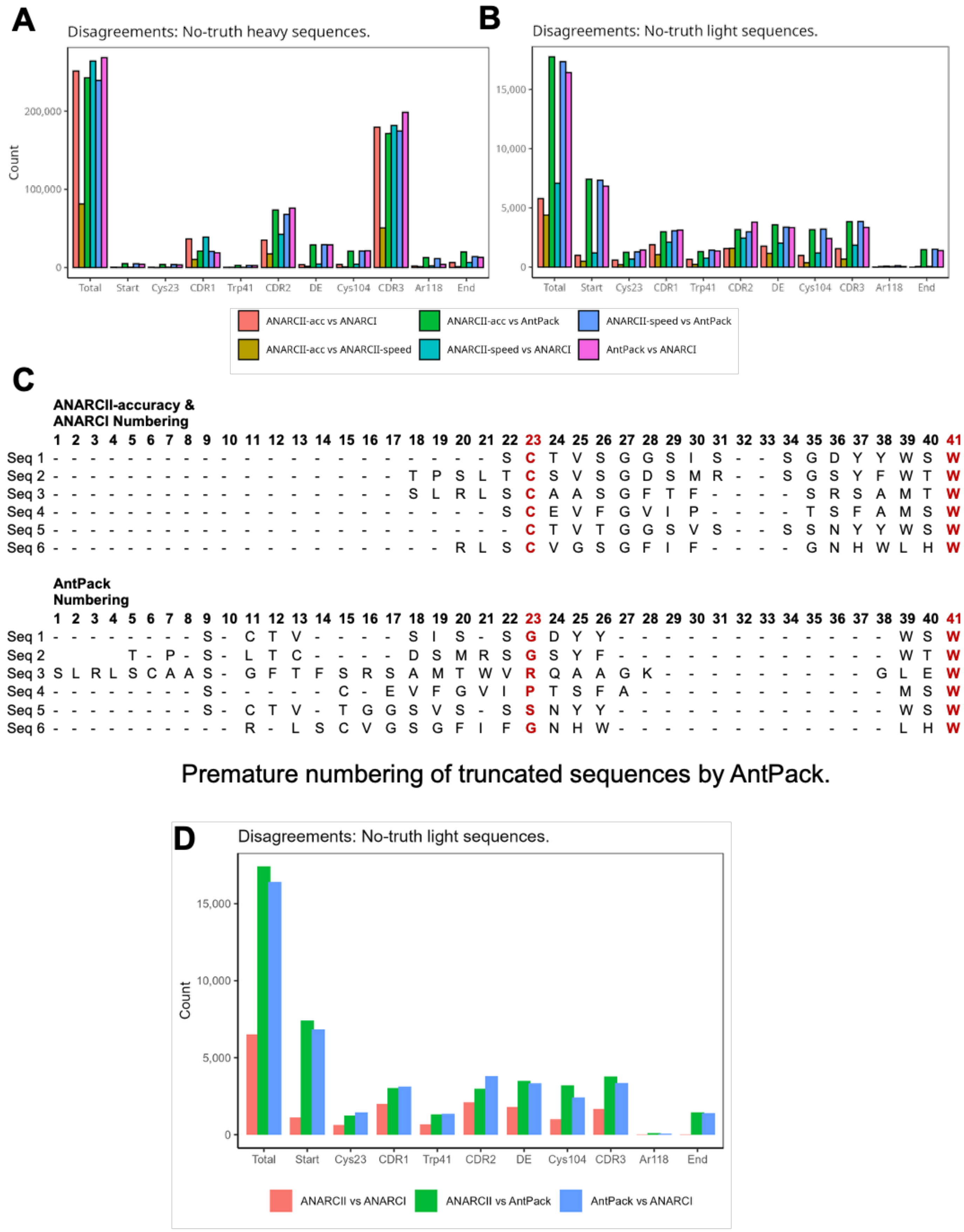

Supplementary Figure 3: Analysis of disagreements between numbering tools on ambiguous sequences. *Comparison of AntPack, ANARCI, ANARCII-accuracy and ANARCII-speed on 'no-truth' heavy (**A**) and light (**B**) sequences (where two versions of ANARCI failed to agree) according to the sequence*

*region where the disagreement occurs. (**C**) Examples of ANARCI/ANARCII-accuracy versus AntPack numbering of truncated sequences corresponding to schematic in Figure 3B. Specifically detailing the early start by AntPack which misses the truncation identified by ANARCI and ANARCII-accuracy. (**D**) Comparison of AntPack, ANARCI, and ANARCII-accuracy on 'no-truth' light sequences (identical to data in part B, however with less comparisons to allow for better readability).*

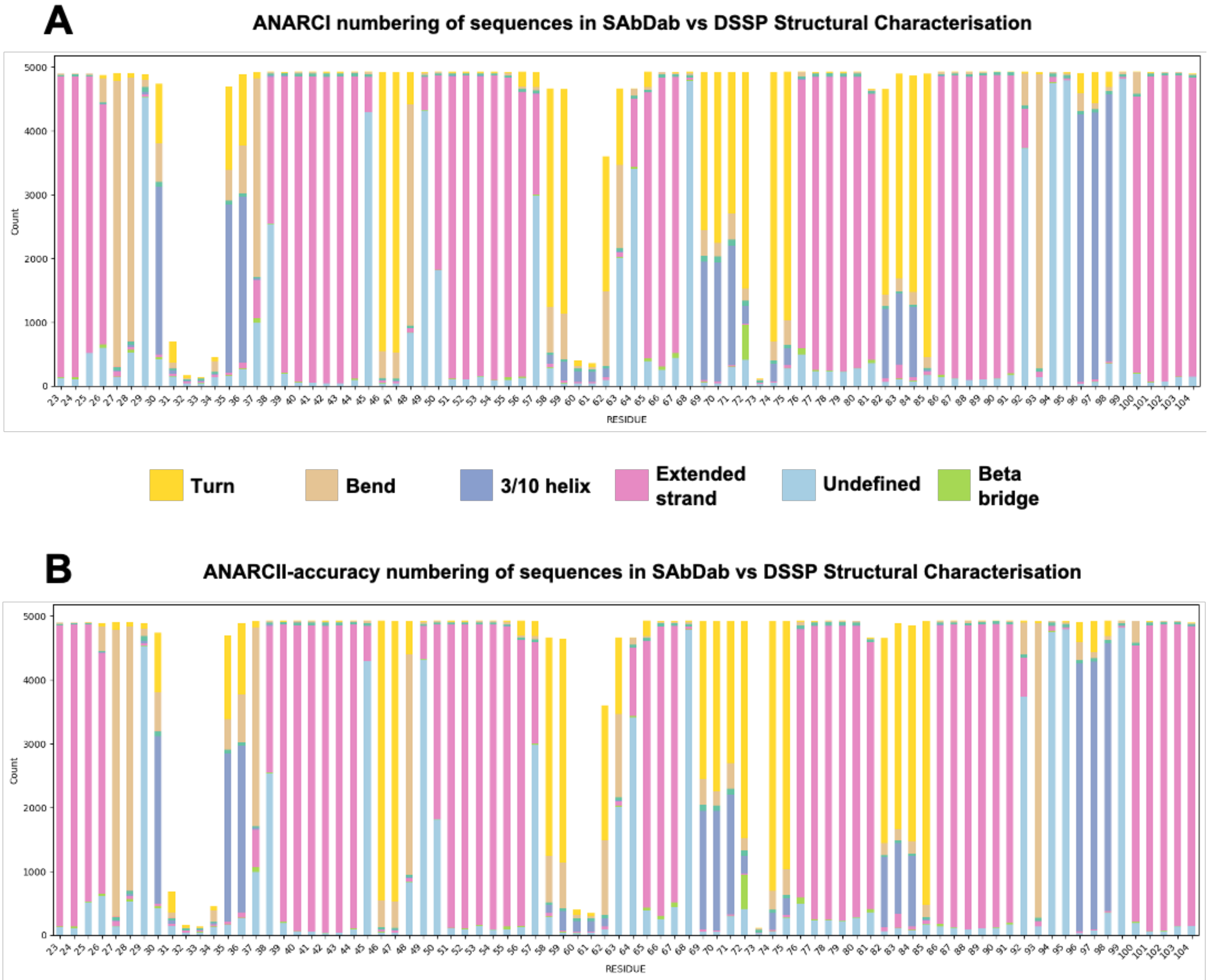

Supplementary Figure 4: Structural analysis of ANARCI and ANARCII-accuracy numbering of sequences in SAbDab. *Dictionary of protein secondary structure (DSSP) characterisation of SAbDab structures numbered with ANARCI (**A**) or ANARCII-accuracy (**B**). Bar plots show the proportion of DSSP classes by IMGT integer position (insertions not shown) in heavy chains for X-ray crystal structures found in SAbDab (one heavy chain analysed for 4945 PDB codes). Clear patterns of secondary structure associated with loops and frameworks align to the assigned IMGT numbering scheme.*

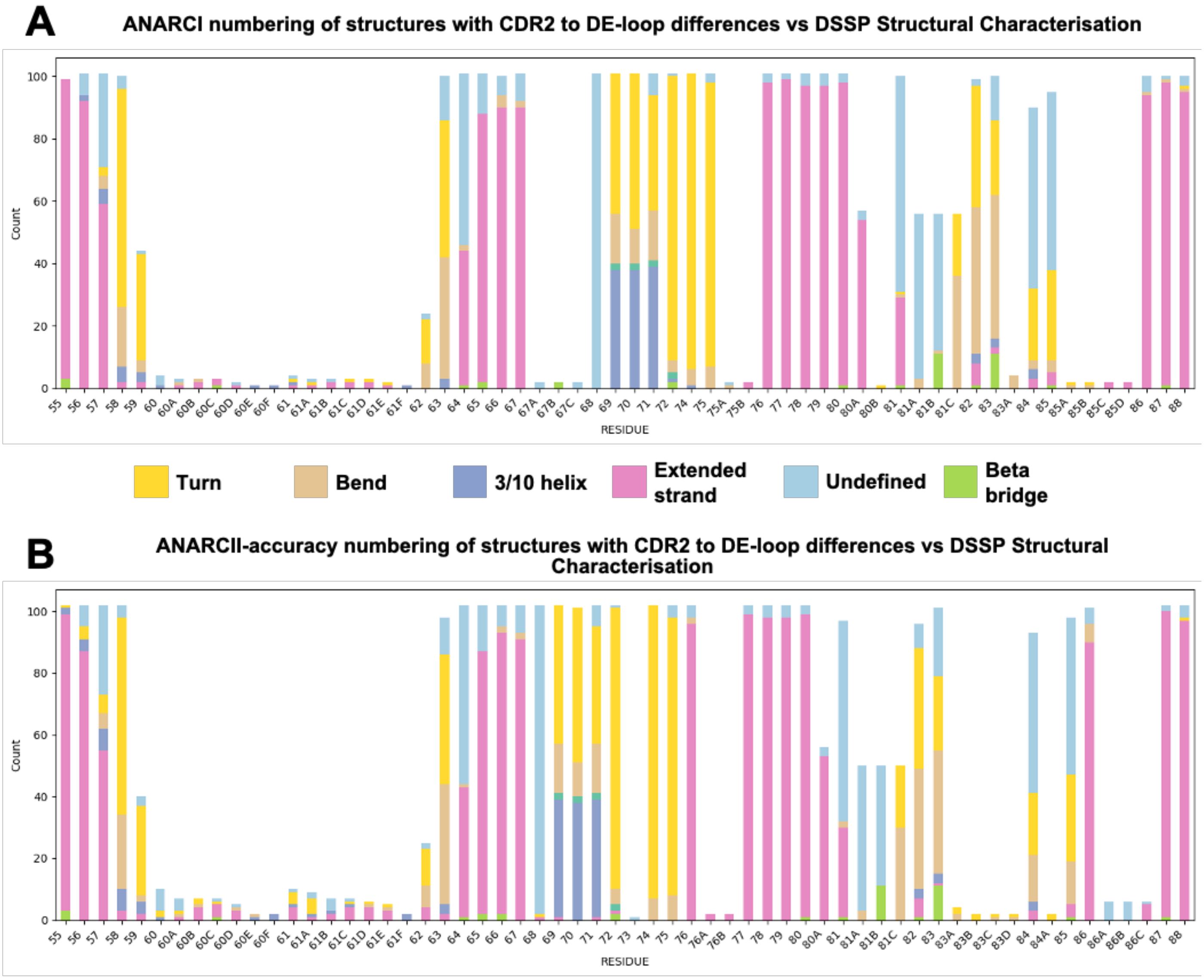

Supplementary Figure 5: Structural analysis of ANARCI and ANARCII-accuracy numbering of sequences in SAbDab which differ in the CDR2 to DE-loop region. *Dictionary of protein secondary structure (DSSP) characterisation of SAbDab heavy chains where the numbering of the CDR2 to DE-loop region disagreed between ANARCI (A) and ANARCII-accuracy (B), corresponding to 113 chains from 57 PDB codes. Bar plots show the proportion of DSSP classes by IMGT position including insertions.*

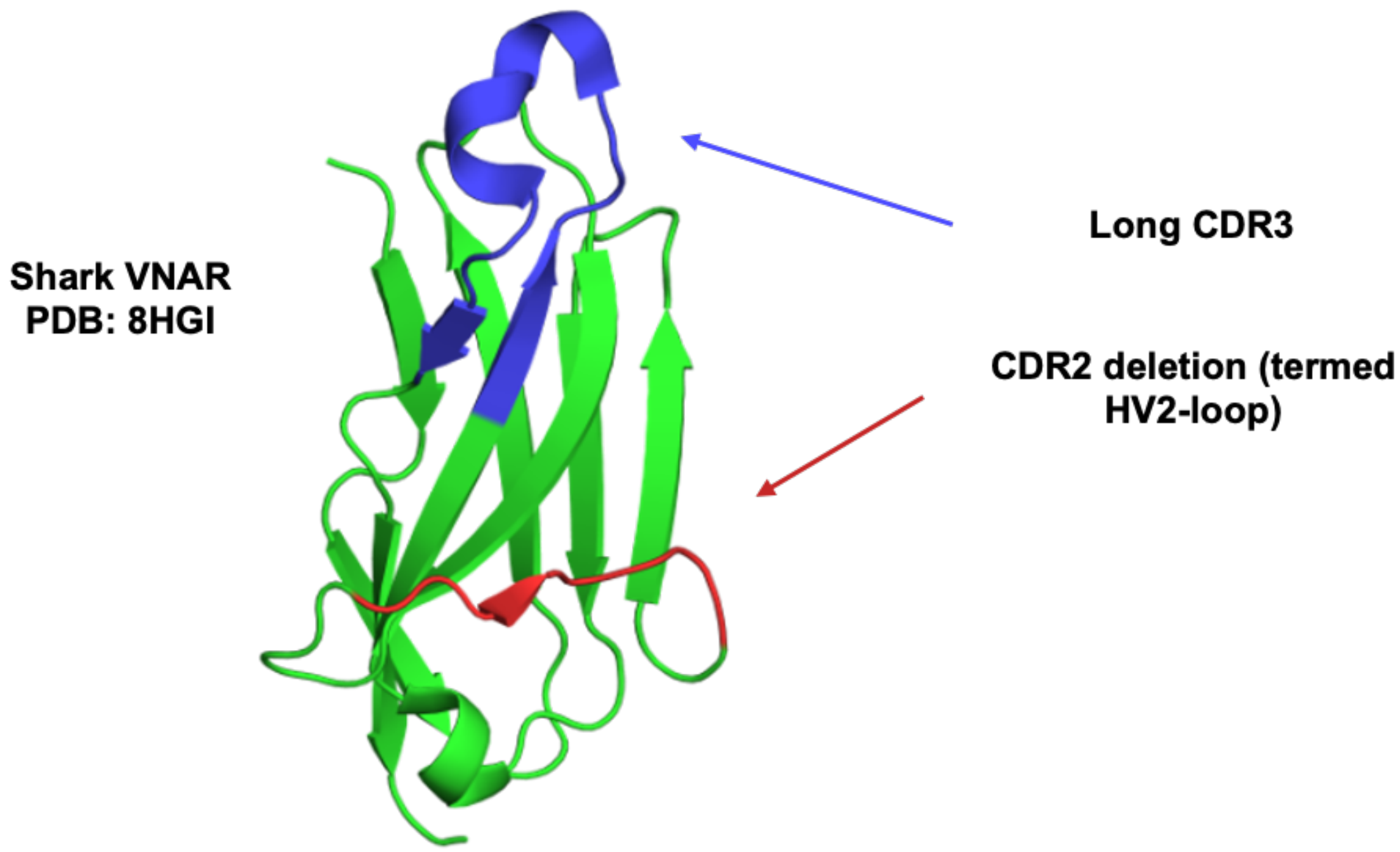

Supplementary Figure 6: Structural features that characterise Shark VNAR sequences. *Example of structural features of shark VNARs which diverge from standard antibody heavy chains. The deletion at the CDR2 (HV2-loop) is shown in red, and long CDR3 shown in blue.*

SUPPLEMENTARY TABLES

Identity	Number of clusters	Singleton clusters	% Singleton clusters	Median size	Mean size	SD	Largest cluster size	Compute time (mins, on 32 CPUs)
50	331	75	23%	6	23,411	153,504	2,283,206	1
55	1,251	354	28%	4	6,194	77,646	2,244,394	1
60	4,454	1,523	34%	3	1,740	39,827	2,169,690	1
65	17,952	6,899	38%	2	432	17,497	1,965,810	5
70	63,076	28,072	45%	2	123	7,437	1,450,012	20
75	173,755	89,162	51%	1	45	3,078	654,086	75
80	405,314	240,280	59%	1	19	1,519	652,127	150
85	782,015	511,462	65%	1	10	806	439,021	260
90	1,243,042	864,621	70%	1	6	451	364,664	165
95	2,053,961	1,539,786	75%	1	4	188	139,087	120

***Supplementary Table 1. Clustering metrics from a random sample of 7.7M heavy chain sequences used to train ANARCII, clustered using CD-HIT at incrementally increasing identity thresholds**. Singleton clusters refer to a cluster comprised of a single sequence.*

	Train Clusters	Train Seqs	Validation Clusters	Validation Seqs	Test Clusters	Test Seqs
Heavy	961,124	73,762,241	53,396	2,723,809	53,396	19,258,862 (random split into 2 subsets)
Kappa	1,654,198	38,715,223	91,900	1,371,630	91,900	1,527,549
Lambda	1,112,239	29,922,215	62,347	1,100,496	62,347	2,359,102

Supplementary Table 2: Numbers of clusters and sequences.

Name	Layers Enc/Dec	Hidden Dims Enc/Dec	Position-wise FF Dims Enc/Dec	Heads	Validation loss after 15 epochs	Best validation loss after 60-100 Epochs	Size (MB)	~Seqs per minute (A100- without optimisation)
Speed	1	128	512	4	0.000860	0.000797	2.2	55,000
Accuracy	2	128	512	4	0.000776	0.000732	4.0	34,000
Super1	1	256	1024	4	0.000791	0.000748	7.9	30,960
Super2	2	256	1024	4	0.000750	0.000716	15.0	18,969

Supplementary Table 3: Model hyperparameters and inference speed (without later optimisations in data processing and value-caching to improve speed).

Name	Chain	Score	Full name	Added To Sabdab
4HGM	K	23.81	4hgm_A mol:protein length:112 Shark V-NAR	TRUE
7SPP	H	20.35	7spp_C mol:protein length:117 VNAR 2C02	TRUE
1VES	H	19.21	1ves_B mol:protein length:113 New Antigen Receptor variable domain	TRUE
8HT3	H	19.14	8ht3_A mol:protein length:104 New Antigen Receptor variable domain	TRUE
7FBK	H	18.9	7fbk_D mol:protein length:129 New antigen receptor variable domain	TRUE
7FBJ	H	18.88	7fbj_N mol:protein length:127 New antigen receptor variable domain	TRUE
2YWZ	H	18.86	2ywz_A mol:protein length:111 new antigen receptor variable domain	TRUE
2Z8V	H	18.82	2z8v_C mol:protein length:116 New antigen receptor variable domain	TRUE
9KQC	H	18.7	9kqc_B mol:protein length:132 VNAR	TRUE
8HGI	H	18.47	8hgi_C mol:protein length:123 VNAR aGFP14	TRUE
4HGK	H	18.38	4hgk_D mol:protein length:128 shark V-NAR antibody	TRUE
1VER	H	18.27	1ver_A mol:protein length:111 New Antigen Receptor variable domain	TRUE
3MOQ	H	18.21	3moq_C mol:protein length:126 New antigen receptor variable domain	TRUE
2Z8W	H	18.17	2z8w_D mol:protein length:116 New antigen receptor variable domain	TRUE
7S83	H	18.14	7s83_B mol:protein length:128 ShAb02 VNAR	TRUE
7SPO	H	17.8	7spo_C mol:protein length:141 VNAR 3B4	TRUE
2I26	H	17.7	2i26_N mol:protein length:121 New Antigen Receptor Ancestral	TRUE
2I27	H	17.7	2i27_N mol:protein length:121 New Antigen Receptor Ancestral	TRUE
5L8K	H	17.67	5l8k_B mol:protein length:117 New antigen receptor variable domain	TRUE
5L8L	H	17.67	5l8l_B mol:protein length:117 New antigen receptor variable domain	TRUE
1T6V	H	17.5	1t6v_O mol:protein length:113 novel antigen receptor	TRUE
1SQ2	H	17.5	1sq2_N mol:protein length:113 novel antigen receptor	TRUE
2YWY	H	17.47	2ywy_A mol:protein length:113 new antigen receptor variable domain	TRUE
2I25	H	17.27	2i25_O mol:protein length:121 New Antigen Receptor PBLA8	TRUE
2I24	H	17.27	2i24_N mol:protein length:121 New Antigen Receptor PBLA8	TRUE
5L8J	H	17.26	5l8j_B mol:protein length:117 New antigen receptor variable domain	TRUE
2COQ	H	17.14	2coq_A mol:protein length:108 new antigen receptor variable domain	TRUE
6X4G	H	16.89	6x4g_N mol:protein length:130 anti ICOS-L VHH domain VNAR	TRUE
3BDB	H	20.49	3bdb_B mol:protein length:137 Novel immune-type receptor 11	FALSE
4NOF	H	18.72	4nof_B mol:protein length:125 Polymeric immunoglobulin receptor	FALSE
2Q3A	L	18.58	2q3a_B mol:protein length:120 CD8	FALSE
7YGG	H	18.56	7ygg_B mol:protein length:142 SIRPA protein	FALSE
2EYR	H	18.35	2eyr_A mol:protein length:210 NKT12	FALSE
2EYS	H	18.35	2eys_A mol:protein length:210 NKT15	FALSE
2EYT	H	18.35	2eyt_A mol:protein length:210 NKT15	FALSE
2QQQ	H	18.13	2qqq_B mol:protein length:111 Novel immune-type receptor 11	FALSE
3B5T	H	18.12	3b5t_C mol:protein length:111 Novel immune-type receptor 10	FALSE
2QTE	H	18.08	2qte_A mol:protein length:111 Novel immune-type receptor 11	FALSE
2QHL	H	18.05	2qhl_E mol:protein length:111 Novel immune-type receptor 10	FALSE
2QJD	H	17.96	2qjd_B mol:protein length:110 Novel immune-type receptor 10	FALSE
4N8P	H	17.8	4n8p_A mol:protein length:135 Uncharacterized protein	FALSE
1HKF	H	17.72	1hkf_A mol:protein length:122 NK CELL ACTIVATING RECEPTOR	FALSE
4KJY	H	17.67	4kjy_B mol:protein length:133 High-affinity SIRPa variant FD6	FALSE
5ELI	K	17.63	5eli_B mol:protein length:126 Triggering receptor expressed on myeloid cells 2	FALSE
6SRU	H	17.49	6sru_F mol:protein length:118 Programmed cell death 1 ligand 1	FALSE
1AH1	H	17.42	1ah1_A mol:protein length:129 CTLA-4	FALSE
6UMT	K	17.35	6umt_A mol:protein length:140 Programmed cell death protein 1	FALSE
4OF6	L	17.16	4of6_B mol:protein length:120 Protein SYG-1	FALSE
4OF7	L	17.16	4of7_A mol:protein length:120 Protein SYG-1	FALSE
2X44	H	17.14	2x44_D mol:protein length:131 CYTOTOXIC T-LYMPHOCYTE PROTEIN 4	FALSE

3J8F	H	17.1	3j8f_7 mol:protein length:423 Poliovirus receptor	FALSE
1BQH	H	17.1	1bqh_I mol:protein length:129 PROTEIN (CD8A OR LYT2 OR LYT-2)	FALSE
5UD8	K	17.09	5ud8_A mol:protein length:112 Triggering receptor expressed on myeloid cells 2	FALSE
4MNQ	H	16.98	4mnq_D mol:protein length:200 Uncharacterized protein	FALSE
3NOI	H	16.74	3noi_A mol:protein length:120 Natural cytotoxicity triggering receptor 3	FALSE
2ICE	H	16.66	2ice_T mol:protein length:119 V-set and immunoglobulin domain-containing protein 4	FALSE
2ICC	H	16.66	2icc_A mol:protein length:119 V-set and immunoglobulin domain-containing protein 4	FALSE
2ICF	H	16.66	2icf_S mol:protein length:119 V-set and immunoglobulin domain-containing protein 4	FALSE
1DR9	H	16.63	1dr9_A mol:protein length:201 T LYMPHOCYTE ACTIVATION ANTIGEN	FALSE
1I8L	H	16.63	1i8l_B mol:protein length:208 T LYMPHOCYTE ACTIVATION ANTIGEN CD80	FALSE
4FMK	L	16.5	4fmk_A mol:protein length:225 Poliovirus receptor-related protein 2	FALSE
4FN0	L	16.5	4fn0_A mol:protein length:225 Poliovirus receptor-related protein 2	FALSE
4NOB	H	16.05	4nob_A mol:protein length:126 Polymeric immunoglobulin receptor	FALSE
6B8O	H	16.03	6b8o_C mol:protein length:169 Triggering receptor expressed on myeloid cells 2	FALSE
5UD7	H	16.03	5ud7_D mol:protein length:169 Triggering receptor expressed on myeloid cells 2	FALSE

Supplementary Table 4: PDB codes identified by ANARCII including VNARS added to SAbDab and examples of non-hypervariable Immunoglobulin variants.

SUPPLEMENTARY REFERENCES

1. Li, W. & Godzik, A. Cd-hit: a fast program for clustering and comparing large sets of protein or nucleotide sequences. *Bioinforma. Oxf. Engl.* **22**, 1658–1659 (2006).
2. Touw, W. G. *et al.* A series of PDB-related databanks for everyday needs. *Nucleic Acids Res.* **43**, D364-368 (2015).
3. Gordon, G. L. *et al.* PLAbDab-nano: a database of camelid and shark nanobodies from patents and literature. *Nucleic Acids Res.* gkae881 (2024) doi:10.1093/nar/gkae881.
