# Supplementary material for: ANARCII enables alignment-free antigen receptor numbering using a generalised language model
Source: Commun Biol. 2026 May 21;9:1085. doi: 10.1038/s42003-026-10186-z (PMC13462589; doi:10.1038/s42003-026-10186-z)
Supplement: Supplementary file 2 [file 42003_2026_10186_MOESM2_ESM.pdf]

Description of Additional Supplementary File

File name: Supplementary Data
Description: Raw data used to generate graphs is available in supplementary data file.
